# Supplementary material for: Bioinspired mechanical mineralization of organogels
Source: Nat Commun. 2023 Dec 14;14:8319. doi: 10.1038/s41467-023-43733-x (PMC10721619; doi:10.1038/s41467-023-43733-x)
Supplement: Supplementary file 1 — Supplementary Information [file 41467_2023_43733_MOESM1_ESM.pdf]

Supplementary Information

Bioinspired mechanical mineralization of organogels

Jorge Ayarza[†,1], Jun Wang[†,1], Hojin Kim[1,2], Pin-Ruei Huang[1], Britteny Cassaidy[1], Gangbin Yan[1], Chong Liu[1], Heinrich M. Jaeger[2,3], Stuart J. Rowan[1,4,5], and Aaron P. Esser-Kahn*[,1]

[†] These authors contributed equally to this work.

*Corresponding author. E-mail: aesserkahn@uchicago.edu

[1] Pritzker School of Molecular Engineering, University of Chicago, 5640 South Ellis Avenue, Chicago, Illinois 60637, United States
[2] James Franck Institute, University of Chicago, 929 East 57th Street, Chicago, Illinois 60637, United States
[3] Department of Physics, University of Chicago, 5720 South Ellis Avenue, Chicago, Illinois 60637, United States
[4] Department of Chemistry, University of Chicago, 5735 South Ellis Avenue, Chicago, Illinois 60637, United States
[5] Chemical and Engineering Sciences Division, Argonne National Laboratory, 9700 Cass Avenue, Lemont, Illinois 60439, United States

Table of Contents

Materials

Instruments

1. Synthetic procedures and characterization
2. NMR spectra
3. Additional characterization of microrods
4. Optimization of the stoichiometry of microrod synthesis
5. Rheology characterization of microrod/PU polymer solution
6. DMA characterization of microrod/azido-PU polymer composites
7. Images of cross-sections of the microrods/azido-PU composite

Supplementary References

Materials

2-mercapto-5-methyl-1,3,4-thiadiazole 95 % (McMT), tris(2-carboxyethyl)phosphine hydrochloride 98 % (TCEP), iodine ≥99.8 % (I_2), poly(propylene glycol) tolylene 2,4-diisocyanate terminated ≥99 % (PPG diisocyanate, M_n 2300 Da, 3.60 w % NCO), bisphenol A diglycidyl ether ≥99 %, triethylenediamine ≥99 %, methylene diphenyl diisocyanate 98 % (MDI), dibutyltin dilaurate 95 % (DBTDL), tetraethylene glycol 99% (TEG), $LiClO_4$ 99.9 %, NaN_3 ≥99.5 % , NaOH 98 %, Na_2SO_4 99 %, $ZnBr_2$ ≥99.9 %, $Zn(NO_3)_2 \cdot 6H_2O$ 98 %, acetonitrile, dimethyl formamide (DMF), dimethyl sulfoxide (DMSO), methanol, dichloromethane, ethyl acetate, n-hexanes, were purchased from Millipore Sigma and used as received, unless otherwise stated. Zinc oxide nanoparticles (ZnO, 99.95 % purity, 18 nm) were purchased from US Research Nanomaterials Inc. Bis(dibenzocyclooctyne-amine) terminated pentaethylene glycol 95 % (DBCO-PEG_5-DBCO) was purchased from Broadpharm. Deuterated solvents were purchased from Cambridge Isotope Laboratories.

Instruments

Chemical characterization

Nuclear magnetic resonance (NMR) spectra (^{1}H and ^{13}C) were recorded either on a Bruker Avance II+ 500 MHz spectrometer equipped with a Bruker QNP probe (^{1}H: 500 MHz, ^{13}C: 125 MHz) or a Bruker Ascend 9.4 T 400 MHz spectrometer equipped with an Avance III HD console and a BBFO “smart” probe (^{1}H: 400 MHz, ^{13}C: 100 Hz) at 25 °C. Chemical shifts (δ) are reported in parts per million (ppm). ^{1}H NMR spectra are referenced to TMS, while ^{13}C NMR spectra are referenced to either TMS or the residual solvent.

High resolution mass spectroscopy (HRMS) was conducted in an Agilent 6224 TOF LC/MS instrument using electrospray ionization (ESI) and a time of flight (TOF) detector.

Fourier transformed infrared (FTIR) spectra were recorded on a Perkin Elmer Spectrum Two FT-IR spectrometer in the MIR range (4000-400 cm^{-1}) using the ATR mode.

X-ray spectra of the microrods were obtained in a KRATOS AXIS NOVA X-ray Photoelectron Spectrometer (XPS) and a Bruker D8 Powder X-ray Diffractometer (XRD).

Gel permeation chromatography (GPC) was conducted in a Tosoh EcoSEC instrument, equipped with Tosoh SuperAW3000 + Tosoh SuperAW4000 + guard columns, eluent: DMF 0.01 M LiBr, flow rate: 0.3 $mL.min^{-1}$, T = 50 ºC and refractive index (RI) and ultraviolet (UV) detectors. The system was calibrated with polystyrene (PS) standards.

Imaging techniques

Scanning electron microscopy (SEM) was conducted in a Carl Zeiss Merlin High-Resolution Field Emission Scanning Electron Microscope (FE-SEM). Samples were previously coated with a Pt film (10 nm thickness) using a Cressington 208 HR sputter coater with Pd/Pt target.

Scanning transmission electron microscopy (STEM) images were acquired using JEOL ARM 200F equipped with a cold field emission source operated at 200 kV. Energy dispersive X-ray (EDS) mapping was acquired using an Oxford X-Max 100TLE windowless SDD detector equipped with JEOL ARM 200F.

Light microscopy images were obtained in a Leica DM2700 P Polarization Microscope under brightfield and polarization modes. A few drops of tetrahydrofuran (THF) were used to swell the polymer composite samples prior to imaging.

Transmission electron microscopy (TEM) was conducted in a ThermoScientific Titan Krios G3i instrument. Cross-sections of polymer composite samples were obtained with a Leica EM UC6 Ultramicrotome.

Thermal and mechanical characterization

Differential scanning calorimetry (DSC) was conducted with a DSC 2500 instrument (TA Instruments) using a modulated DSC program in the range -90 to 250 ºC.

Rheological measurements were conducted with a rotational rheometer (TA Instruments, ARES G2) equipped with a peltier element and a parallel plate fixture (25 mm). A volume of 100 µL of sample was used for each measurement, with a measuring gap of 250 µm, and the temperature set at 25 ºC.

Dynamic mechanical analysis (DMA) was conducted with a TA Instruments RSA-G2 DMA using the compression mode parallel disc fixture (25 mm).

1. Synthetic procedures and characterization

Representative synthesis of the microrods

In a cylindrical polypropylene vial, McMT (66 mg, 0.50 mmol) was dissolved in DMF (400 μL). ZnO nanoparticles (20 mg, 0.25 mmol) were added to the solution and homogenously dispersed via sonication for 20 s. The mixture was sonicated (40 kHz) in the dark for 4 h. The product was diluted with methanol (5 mL) and separated by centrifugation (3000 x g for 10 min). The precipitate was washed twice with methanol (10 mL) and again separated by centrifugation. The product was dried at 50 ºC under vacuum overnight. Yield: 71 mg, 86 %.

Synthesis of Zn(McMT)$_n$ complex [1]

A solution of $Zn(NO_3)_2 \cdot 6H_2O$ (149 mg, 0.50 mmol) in water (1 mL) was prepared. In a separate vial, McMT (132 mg, 1.00 mmol) was dissolved in a $NaOH_{(aq)}$ 1 M solution (1 mL). The zinc nitrate solution was added dropwise to the McMT solution under vigorous stirring. A white precipitate formed, and the mixture was stirred for 30 min. The solid was recovered by filtration and washed thoroughly with water. The product was dried at 50 ºC under vacuum overnight. Yield: 128 mg, 78 %.

Synthesis of McMT disulfide dimer (2-methyl-5-[(5-methyl-1,3,4-thiadiazol-2-yl)disulfanyl]-1,3,4-thiadiazole) [2]

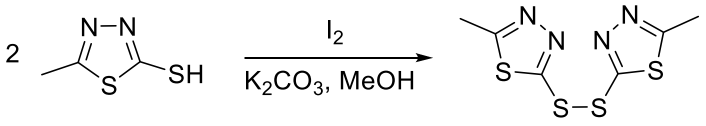

McMT (1.32 g, 10 mmol) was dissolved in 20 mL of methanol and K_2CO_3 (1.38 g, 10 mmol) was added. The mixture was placed in an ultrasound bath for 10 min and then stirred for 1 h. Then, iodine (1.27 g, 5 mmol) was added, and the mixture was stirred for 1 h at room temperature. The product was concentrated under vacuum and diluted with 20 mL of dichloromethane. The solution was washed with water (3 x 50 mL), brine (1 x 50 mL), and finally the organic phase was dried with anhydrous Na_2SO_4. The organic phase was concentrated under vacuum, resulting in a white solid. The product was dried under a high vacuum overnight. Yield: 1.15 g, 88 %. **^{1}H NMR** (500 MHz, $CDCl_3$): δ_H (ppm) 2.78 (s, 6H, CH_3). **^{13}C NMR** (125 MHz, $CDCl_3$): δ_C (ppm) 168.9 (S**C**(N)S), 166.1 (H_3C**C**(N)S), 16.0 (CH_3).

Control reactions

w/ respect to representative procedure for the synthesis of microrods

- No ZnO: sample without ZnO nanoparticles.
- No US: sample was stirred in a vortex at 100 rpm.
- w/ $ZnBr_2$: instead of ZnO nanoparticles, anhydrous $ZnBr_2$ (56 mg, 0.25 mmol) was added and dissolved in the reaction mixture.
- w/ McMT disulfide dimer: instead of McMT, the disulfide (66 mg, 0.13 mmol) was added and dissolved in the reaction mixture.

Synthesis of microrods within polymer organic solution

In a cylindrical polypropylene vial, poly(propylene glycol) tolylene 2,4-diisocyanate terminated (PPG diisocyanate, M_n 2300 Da, 3.60 w % NCO, 0.50 g, 0.43 mmol NCO) was weighed. PPG diisocyanate was dissolved in DMF (1 mL) and 21 mg of tetraethylene glycol (0.11 mmol glycerol, 0.22 mmol OH) were added. Dibutyltin dilaurate (3 µL) was added to the mixture and the solution was vortexed for 30 s. Then, McMT (132 mg, 1.00 mmol) was added, and the mixture was vortexed until dissolution. Finally, ZnO nanoparticles (40 mg, 0.50 mmol) were added and dispersed under sonication. The mixture was sonicated in an ultrasound bath (40 kHz) for 6 h.

*Synthesis of Bisphenol A di(3-azido-2 hydroxy propan-1-ol) ether (**BPA-diazide**)* [3]

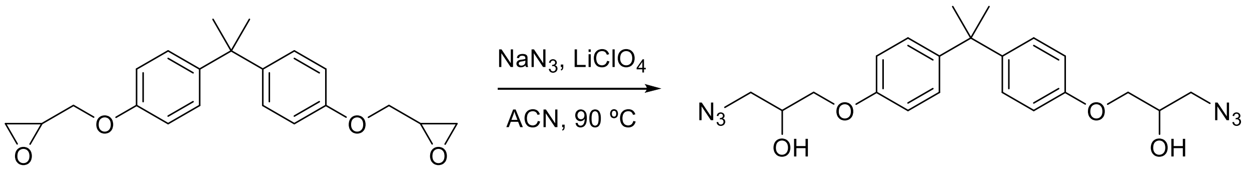

A solution of bisphenol A diglycidyl ether (16.8 g, 49.5 mmol) and $LiClO_4$ (16.6 g, 157 mmol) in acetonitrile (150 mL) was prepared. NaN_3 (10.0 g, 154 mmol) was added in portions and the reaction mixture was heated to 90 ºC under Ar atmosphere with vigorous stirring for 24 h. Afterwards, the reaction was cooled down to room temperature and water (300 mL) was added. The reaction mixture was extracted with ethyl acetate (3 × 150 mL), and the combined organic layer was washed with water (3 × 100 mL) and brine (3 × 100 mL), dried over anhydrous Na_2SO_4 and concentrated *in vacuo* to yield a light-yellow viscous oil. The product was further purified by flash column chromatography using an ISCO Teledyne instrument with a 330 g RediSep Rf silica column while eluting with a mixture of hexanes and ethyl acetate (gradient of 0 to 40 % ethyl acetate). Fractions were combined and concentrated *in vacuo*, yielding a pale viscous oil of bisphenol A di(3-azido-2 hydroxy propan-1-ol) ether (15.8 g, 37.1 mmol, yield 75 %).

^{1}H NMR (500 MHz, $(CD_3)_2SO$): δ_H (ppm) 7.10 (d, 4H, $^3J_{H-H}$ = 8.7 Hz, Ar-$C_{3,5}$-H), 6.83 (d, 4H, $^3J_{H-H}$ = 8.7 Hz, Ar-$C_{2,6}$-H), 5.51 (d, 2H, $^3J_{H-H}$ = 5 Hz, OH), 3.99 (m, 2H, CH), 3.88 (m, 4H, O-CH_2), 3.35 (m, 4H, CH_2-N_3), 1.57 (s, 6H, CH_3). **^{13}C NMR** (125 MHz, $CDCl_3$): δ_C (ppm) 156.0 (Ar-C_1),

143.8 (Ar-C_4), 127.8 (Ar-$C_{3,5}$), 113.9 (Ar-$C_{2,6}$), 69.3 (CH), 69.0 (O-CH_2), 53.3 (CH_2-N_3), 41.7 (C_{quat}), 30.9 (CH_3). **HRMS** ESI-TOF (m/z): $[M+Cl]^-$ calcd. for $C_{21}H_{26}N_6O_4Cl$: 461.1704 found: 461.1685.

Synthesis of azido-PU [3]

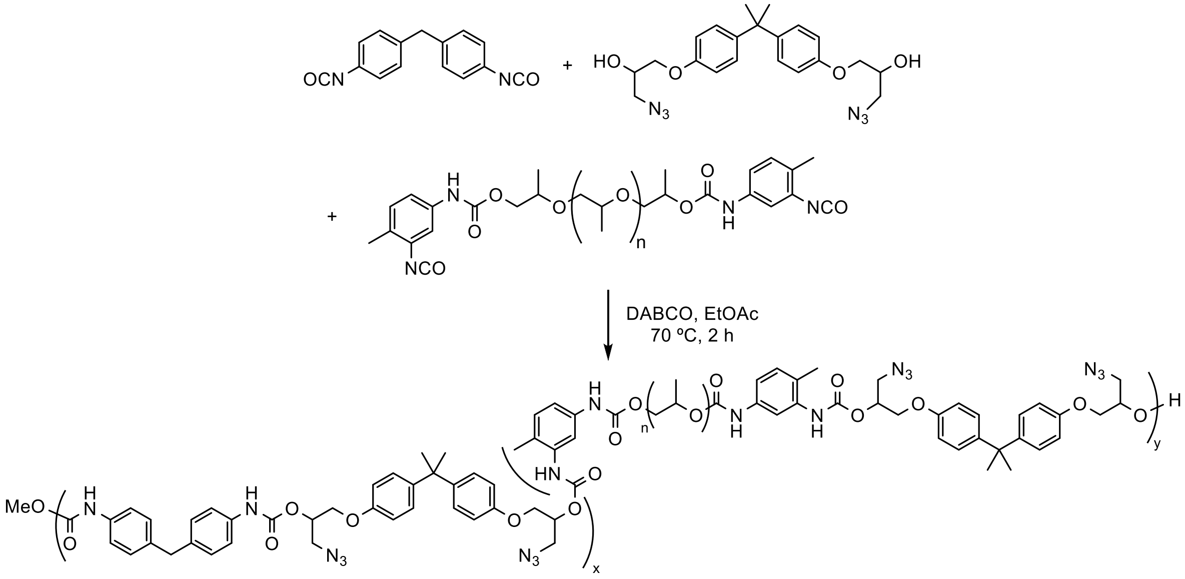

In a round-bottom flask, poly(propylene glycol) tolylene 2,4-diisocyanate terminated (PPG diisocyanate, M_n 2300 Da, 3.60 w % NCO, 1.35 g, 0.59 mmol) was mixed with methylene diphenyl diisocyanate (440 mg, 1.76 mmol) and dissolved in ethyl acetate (2 mL). Separately, in a vial, BPA-diazide (1.00 g, 2.35 mmol) was mixed with triethylenediamine (28 mg, 0.25 mmol) and dissolved in ethyl acetate (2 mL). The second solution was added to the first one and the mixture was homogenized under vigorous stirring, followed by heating at 70 ºC for 2 h. After cooling the reaction mixture to room temperature, it was diluted with ethyl acetate (4 mL) and precipitated in cold hexanes (100 mL). The polymer was recovered by centrifugation, washed overnight in hexanes (50 mL), and dried under vacuum at 60 ºC overnight. Yield: 2.68 g, 96 %. **^{1}H NMR** (400 MHz, $(CD_3)_2SO$): δ_H (ppm) 9.78 (s), 9.53 (s), 9.04 (s), 7.36 (d), 7.08 (d), 6.83 (d), 5.52 (d), 5.18 (t), 4.86 (m), 4.12 (d), 3.87 (t), 3.79 (s), 3.67-3.71 (m), 3.41-3.48 (m), 2.71 (s), 2.10 (s), 1.56 (s), 1.19 (t), 1.04 (s), 1.02 (s).

Synthesis of polymer organogel with embedded microrods

In a cylindrical polypropylene vial, azido-PU (150 mg) was dissolved in DMF (400 μL) overnight. Separately, in a vial, DBCO-PEG_5-DBCO (15 mg) was dissolved in DMF (50 μL). McMT (66 mg) was added to the azido-PU solution and dissolved by vigorous stirring. ZnO nanoparticles (20 mg) were added and homogenously dispersed by vigorous stirring and sonication for 20 s. The mixture was cooled down in a fridge (4 ºC) for 30 min. The crosslinker solution was quickly added to the azido-PU mixture and rapidly vortexed. The organogel forms within 1 min. It was then sonicated in an ultrasound bath (40 kHz) for 6 h. The organogel was then carefully removed from the vial and dried in a vacuum oven (50 ºC) overnight to remove the DMF.

2. NMR spectra

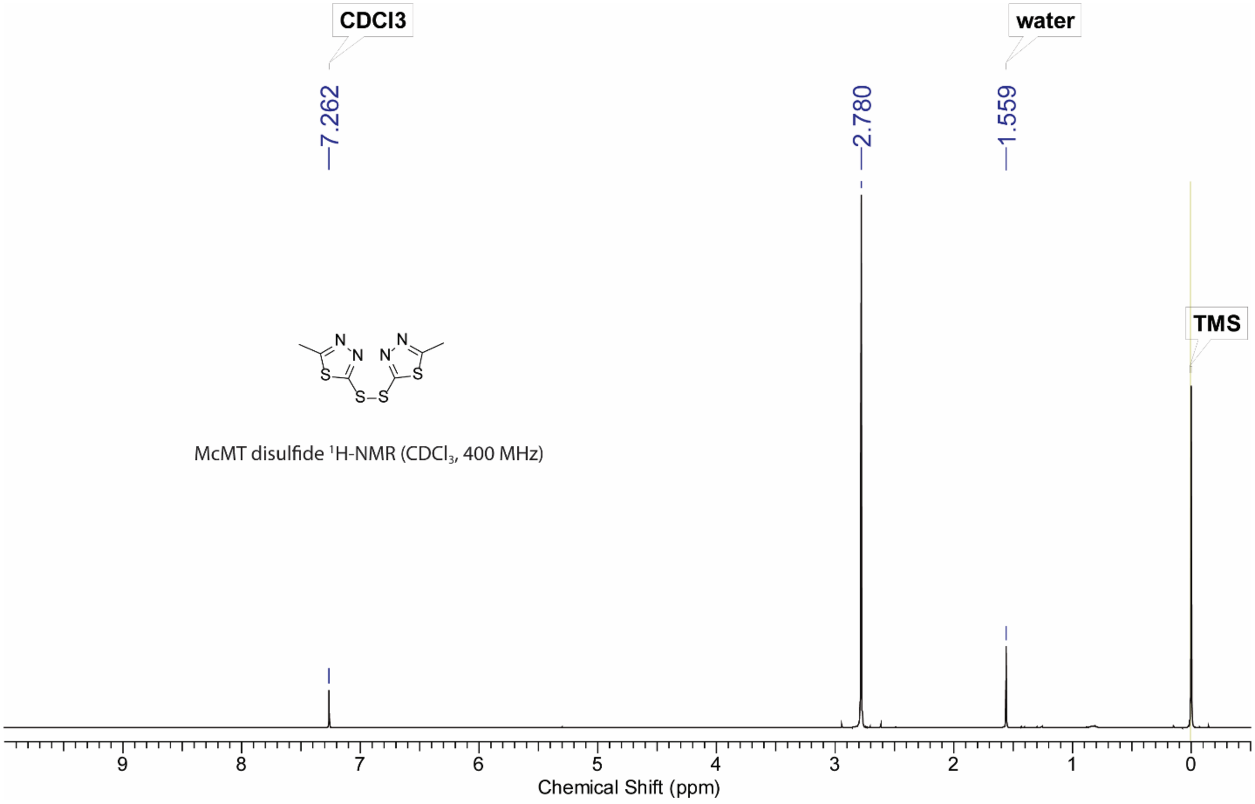

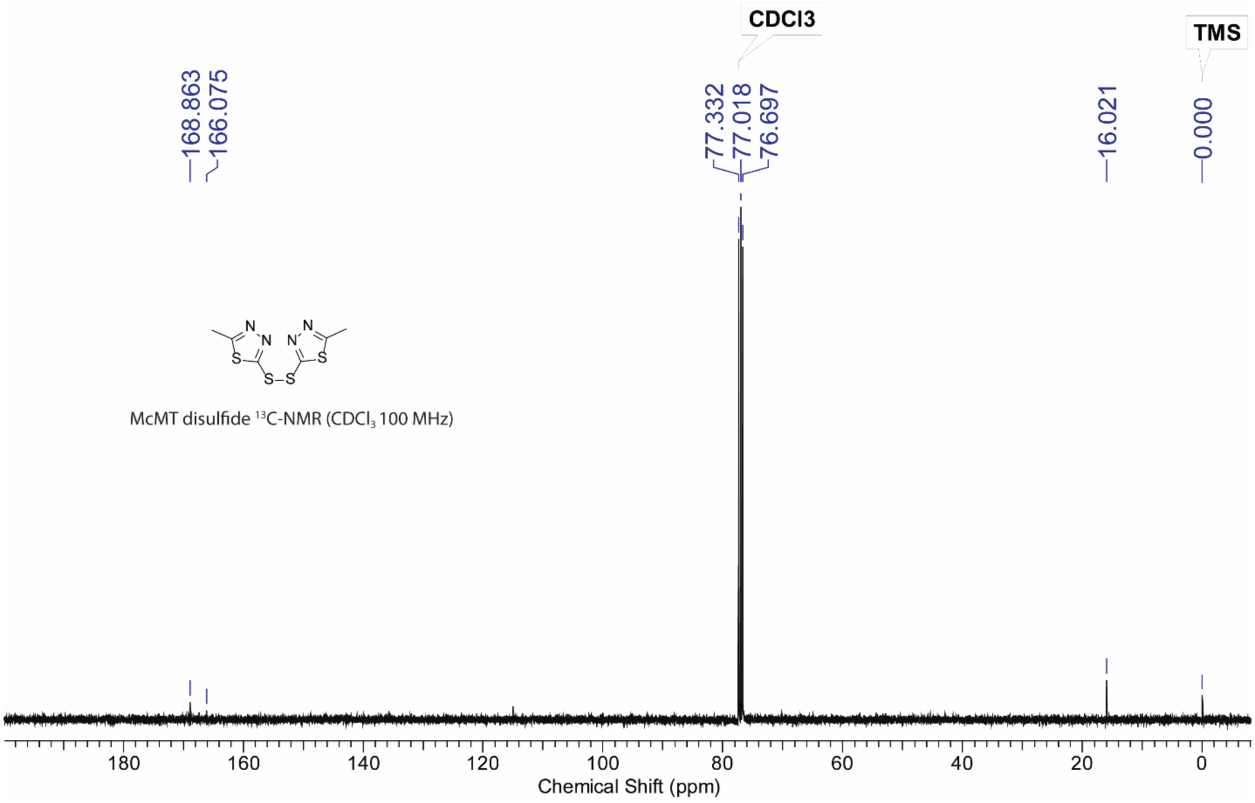

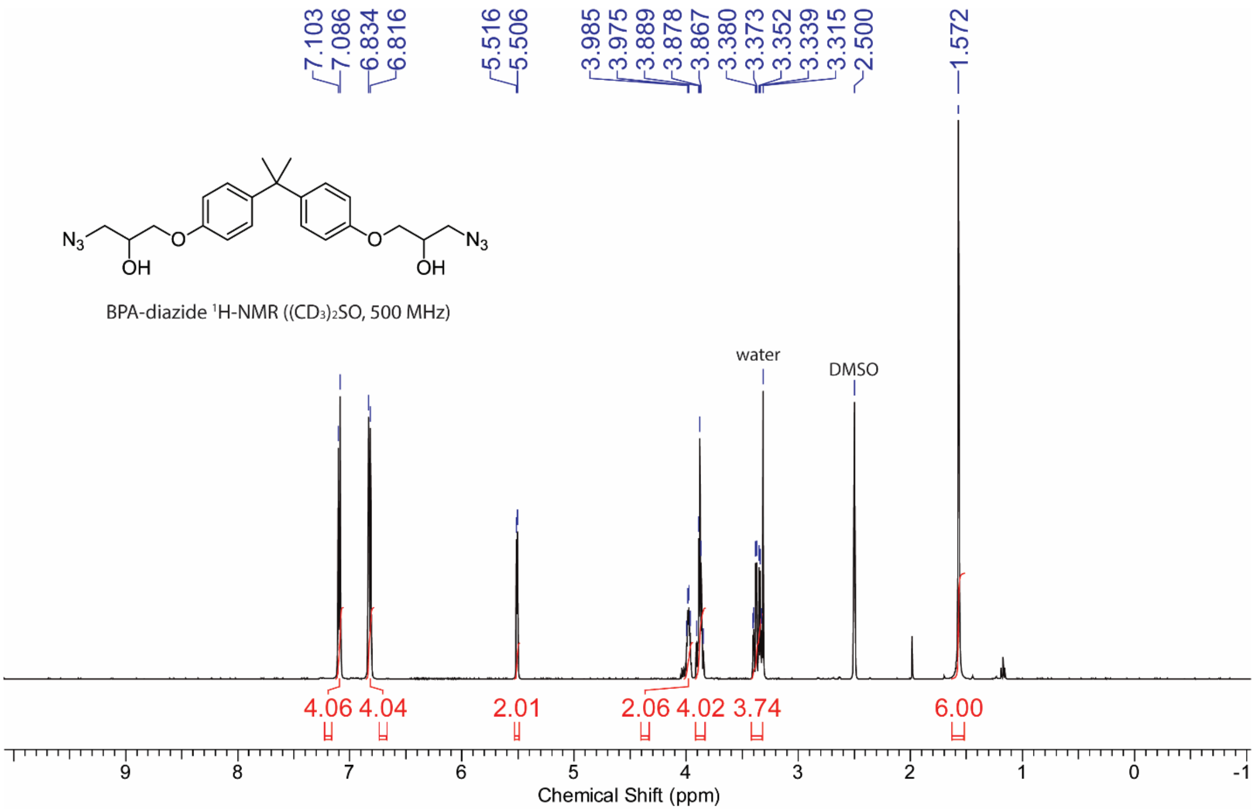
7.103
7.086
6.834
6.816
5.516
5.506
3.985
3.975
3.889
3.878
3.867
3.380
3.373
3.352
3.339
3.315
2.500
1.572
N_3
OH
O
O
OH
N_3
BPA-diazide ^{1}H-NMR ((CD$_3$)$_2$SO, 500 MHz)
water
DMSO
4.06 4.04
2.01
2.06 4.02 3.74
6.00
9 8 7 6 5 4 3 2 1 0 -1
Chemical Shift (ppm)

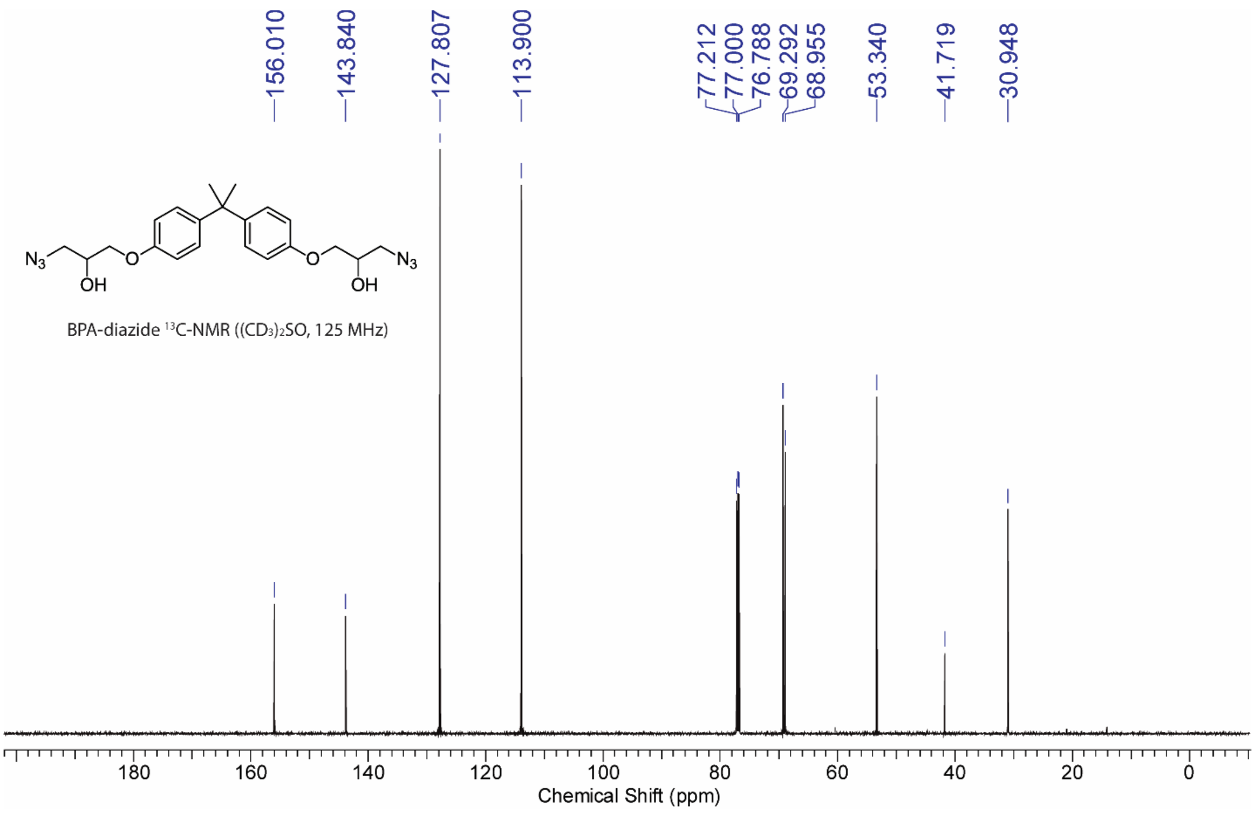
156.010
143.840
127.807
113.900
77.212
77.000
76.788
69.292
68.955
53.340
41.719
30.948
N_3
OH
O
O
OH
N_3
BPA-diazide ^{13}C-NMR ((CD$_3$)$_2$SO, 125 MHz)
180 160 140 120 100 80 60 40 20 0
Chemical Shift (ppm)

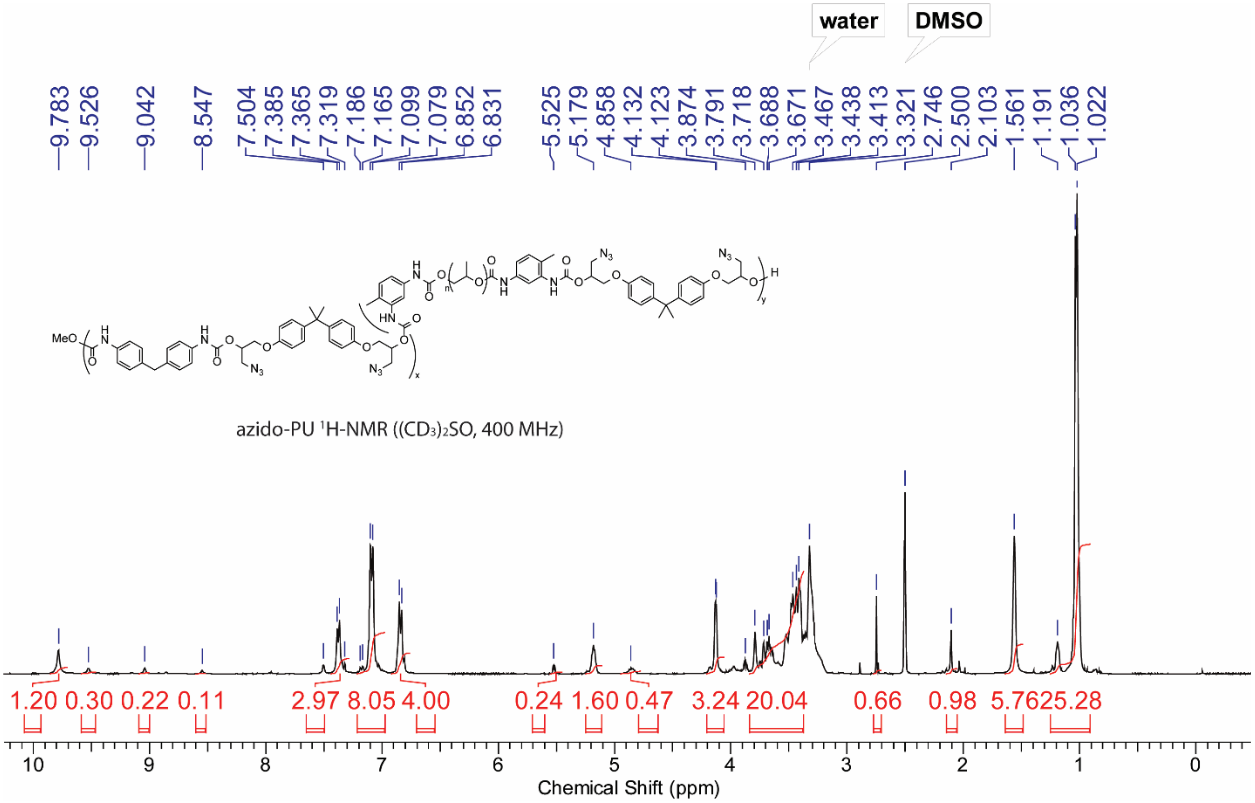
water
DMSO
9.783
9.526
9.042
8.547
7.504
7.385
7.365
7.319
7.186
7.165
7.099
7.079
6.852
6.831
5.525
5.179
4.858
4.132
4.123
3.874
3.791
3.718
3.688
3.671
3.467
3.438
3.413
3.321
2.746
2.500
2.103
1.561
1.191
1.036
1.022
azido-PU ¹H-NMR ((CD₃)₂SO, 400 MHz)
1.20
0.30
0.22
0.11
2.97
8.05
4.00
0.24
1.60
0.47
3.24
20.04
0.66
0.98
5.76
25.28
Chemical Shift (ppm)

3. Additional characterization of microrods

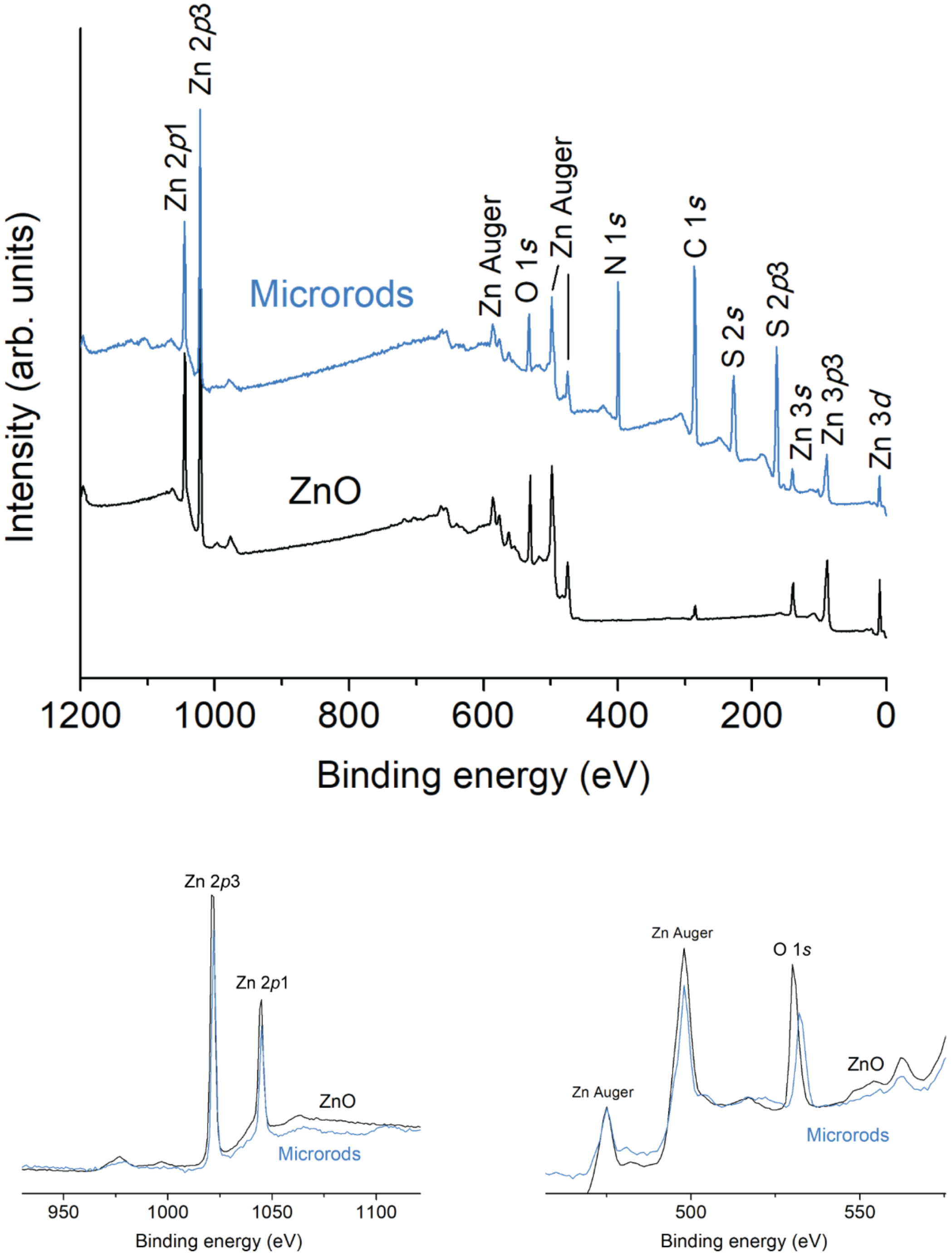

Supplementary Fig. 1. Graph comparing the XPS spectra of ZnO and microrods (*top*), with close-up images of the Zn and O peaks (*bottom*).

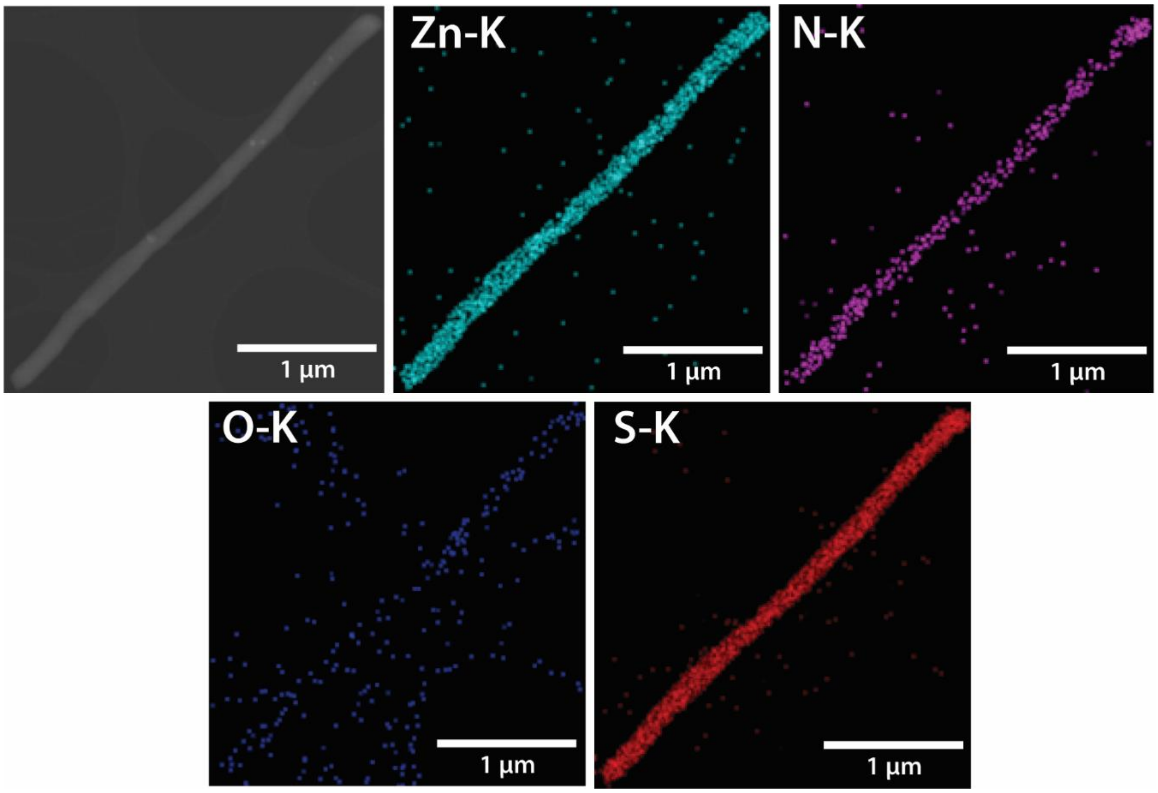

Supplementary Fig. 2. STEM images of the microrods and EDS elemental mapping.

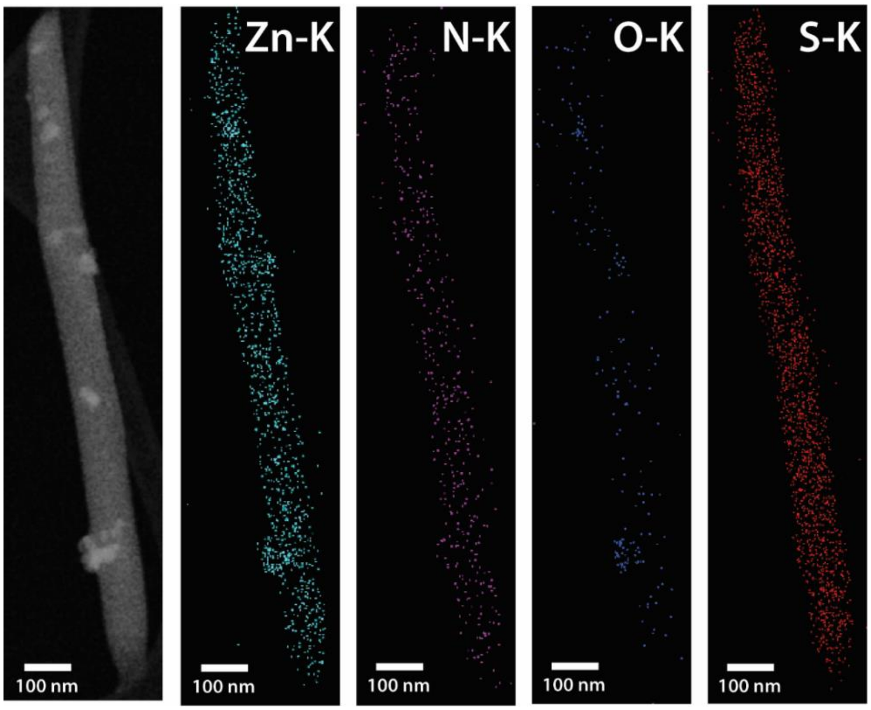

Supplementary Fig. 3. STEM images of the Zn(McMT)$_n$ complex and EDS elemental mapping.

Atlantic Microlab, Inc.

Sample No. PRH-JA-Sn

6180 Atlantic Blvd. Suite M
Norcross, GA 30071
www.atlanticmicrolab.com

C6 H8 N4 S4 Zn
329.78

Company/School University of Chicago
Dept. Pritzker School of Molecular Engingeering
Address 5640 S Ellis Ave
City, State, Zip Chicago, IL 60637
Name [redacted] Date 08/03/2023
Phone [redacted]

Professor/Supervisor: Aaron Esser-Kahn
PO# / CC#

Element	Theory	Found	
C	21.85	21.12	21.21
H	2.45	1.72	1.70
N	16.99	16.22	16.40
S	38.89	37.58	37.49

Single ☐ Duplicate ☒
Elements Present: C H N S Zn O(Possible)
Analyze for: C H N S
Hygroscopic ☐ Explosive ☐
M.P. ≥200 Celcius B.P. unknown
To be dried: Yes ☒ No ☐
Temp. 50C Vac. yes Time 30min
Rush Service ☒ Rush service guarantees analyses will be completed and results available by 5 PM EST on the day the sample is received by 11 AM.
Include Email Address or FAX # Below
huangpr@uchicago.edu

Date Received AUG 0 9 2023 Date Completed AUG 0 9 2023
Remarks:

Supplementary Fig. 4. Elemental analysis of the microrods.

Atlantic Microlab, Inc.

Sample No. PRH-JA-Thermal

6180 Atlantic Blvd. Suite M
Norcross, GA 30071
www.atlanticmicrolab.com

C6 H8 N4 S4 Zn
329.78

Company/School University of Chicago
Dept. Pritzker School of Molecular Engingeering
Address 5640 S Ellis Ave
City, State, Zip Chicago, IL 60637
Name [redacted] Date 08/03/2023
Phone [redacted]

Professor/Supervisor: Aaron Esser-Kahn
PO# / CC#

Element	Theory	Found	
C	21.85	19.37	19.34
H	2.45	1.55	1.46
N	16.99	14.85	14.81
S	38.89	33.92	33.83

Single ☐ Duplicate ☒
Elements Present: C H N S Zn O(Possible)
Analyze for: C H N S
Hygroscopic ☐ Explosive ☐
M.P. ≥200 Celcius B.P. unknown
To be dried: Yes ☒ No ☐
Temp. 50C Vac. yes Time 30min
Rush Service ☒ Rush service guarantees analyses will be completed and results available by 5 PM EST on the day the sample is received by 11 AM.
Include Email Address or FAX # Below
huangpr@uchicago.edu

Date Received AUG 0 9 2023 Date Completed AUG 0 9 2023
Remarks:

Supplementary Fig. 5. Elemental analysis of the Zn(McMT)$_n$ complex.

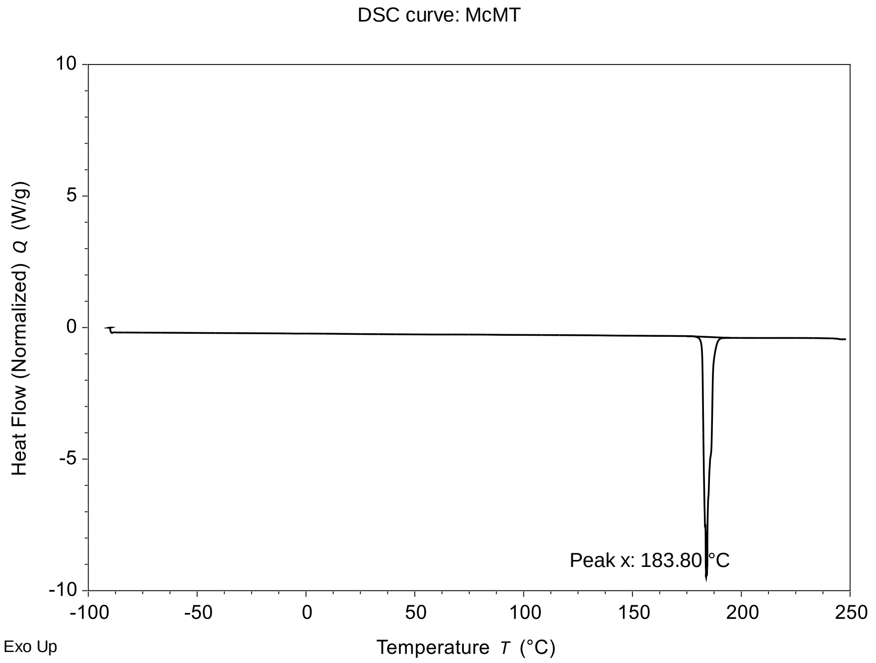

Supplementary Fig. 6. Modulated DSC curve of McMT.

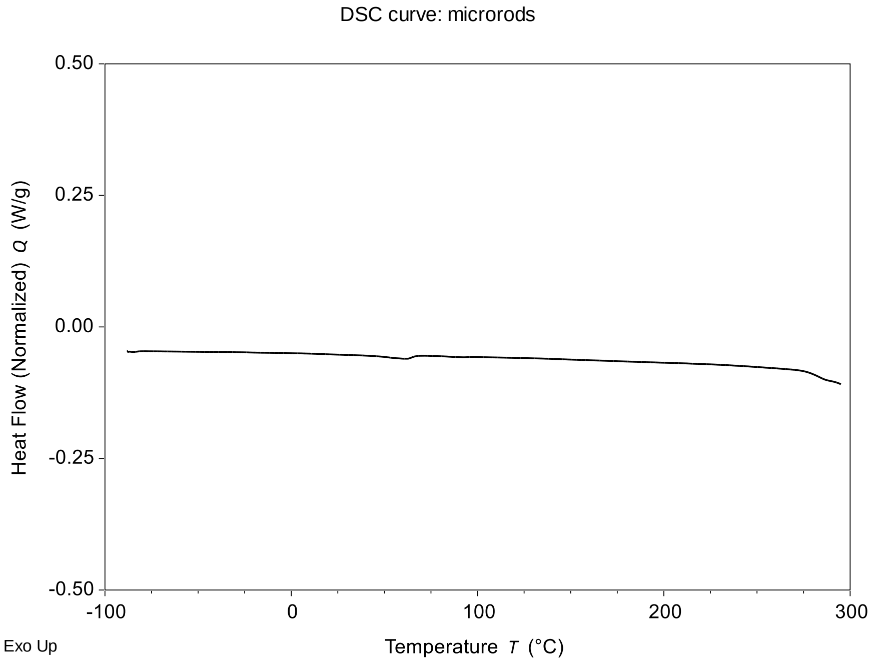

Supplementary Fig. 7. Modulated DSC curve of microrods.

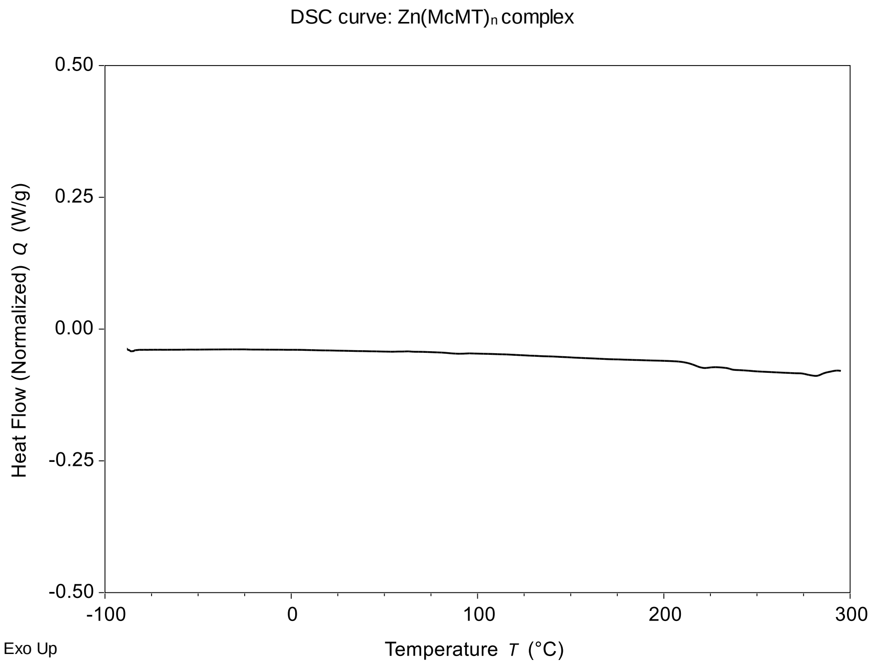

Supplementary Fig. 8. Modulated DSC curve of $Zn(McMT)_n$ complex.

4. Optimization of the stoichiometry of microrod synthesis

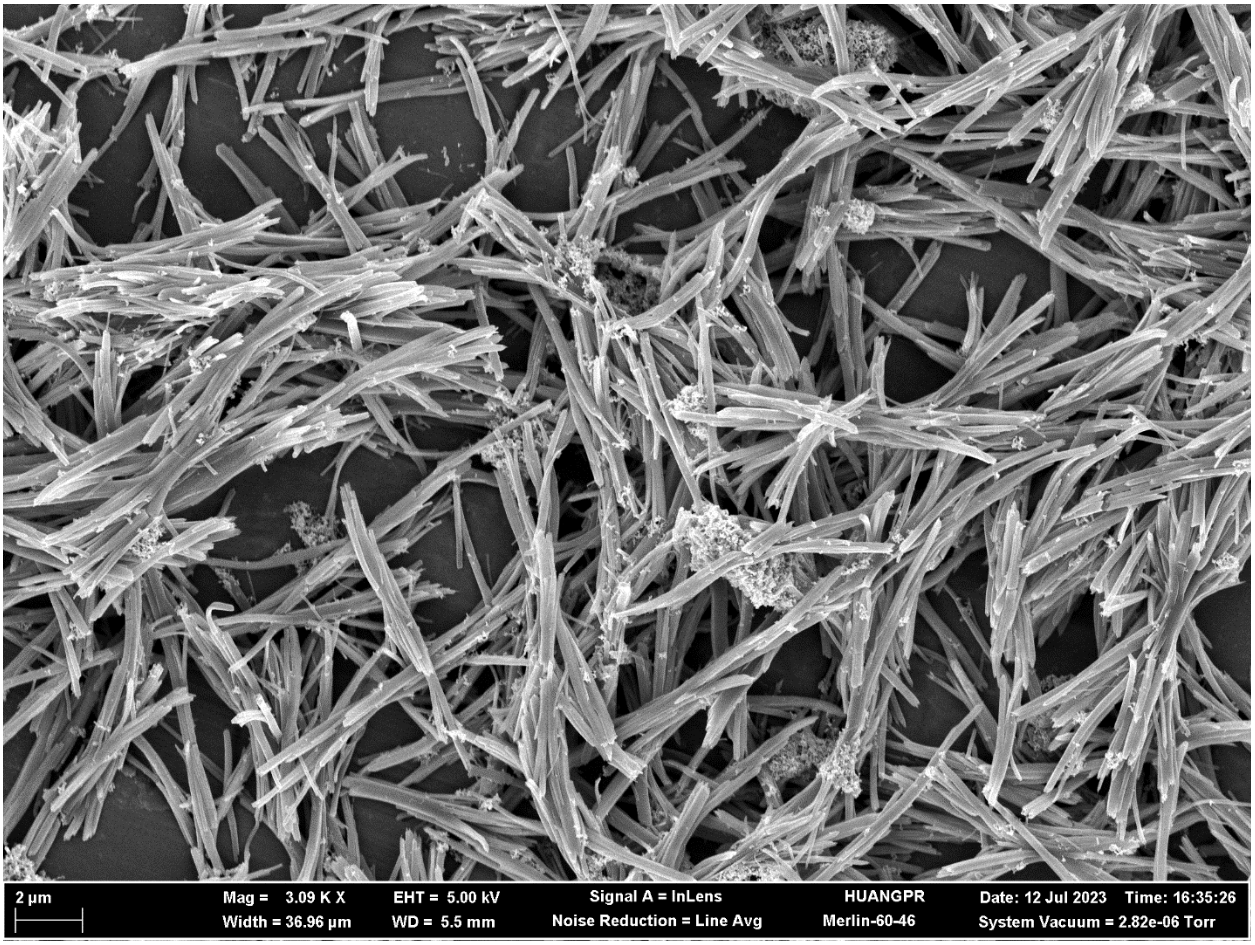

Supplementary Fig. 9. SEM image of the microrods obtained via mechanical stirring with a magnetic stir bar (400 rpm). Average length (3.9 µm) and width (0.3 µm).

Supplementary Table 1. Results for experiments varying the concentration of ZnO.

Sample	ZnO (mmol)	McMT (mmol)	Molar ratio (ZnO:McMT)	Yield (mg)
ZnO (10 mg)	0.13	0.50	1:4	33
ZnO (20 mg)	0.25	0.50	1:2	70
ZnO (40 mg)	0.50	0.50	1:1	93*

*The increase in yield can be attributed to the unreacted ZnO which is also recovered during the microrod isolation procedure.

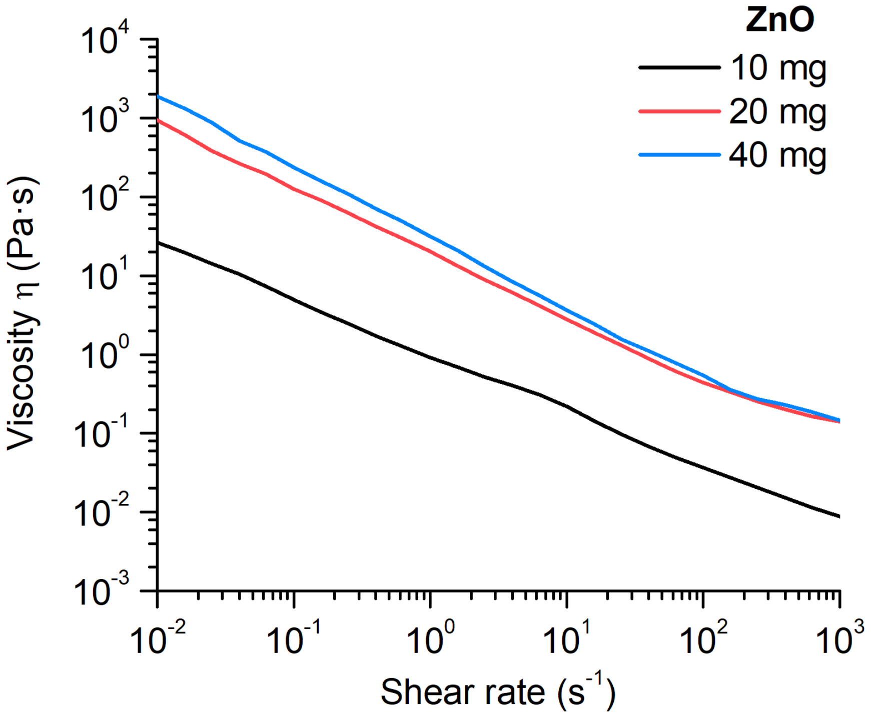

Supplementary Fig. 10. Flow viscosity measurements of the microrod suspensions resulting from reactions varying the concentration of ZnO.

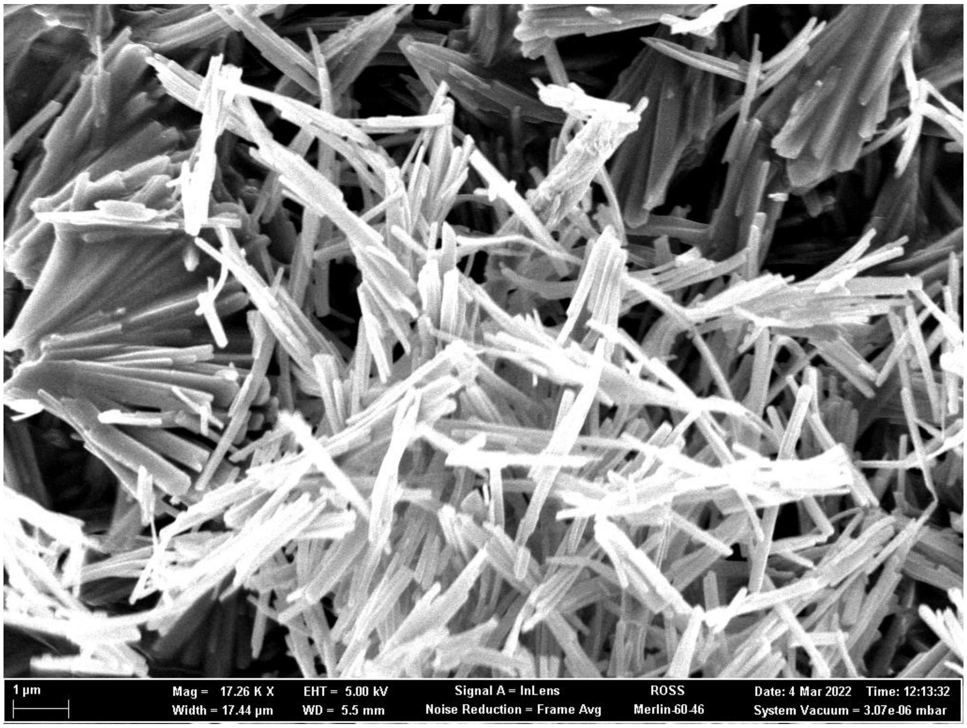

Supplementary Fig. 11. SEM image of microrods from sample with ZnO (10 mg).

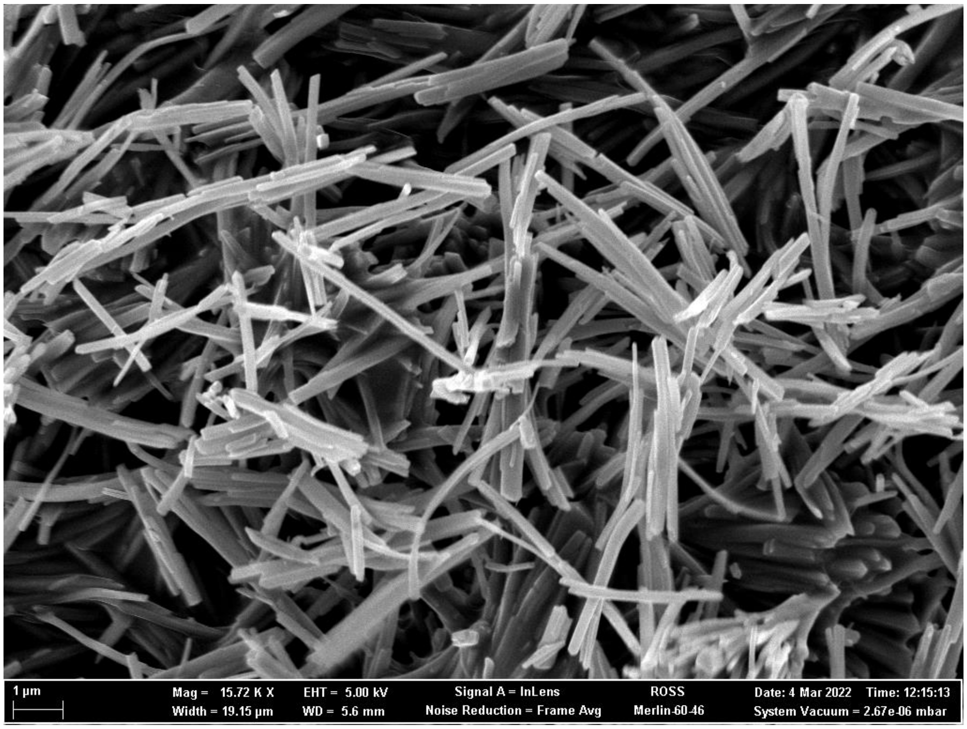

Supplementary Fig. 12. SEM image of microrods from sample ZnO (20 mg).

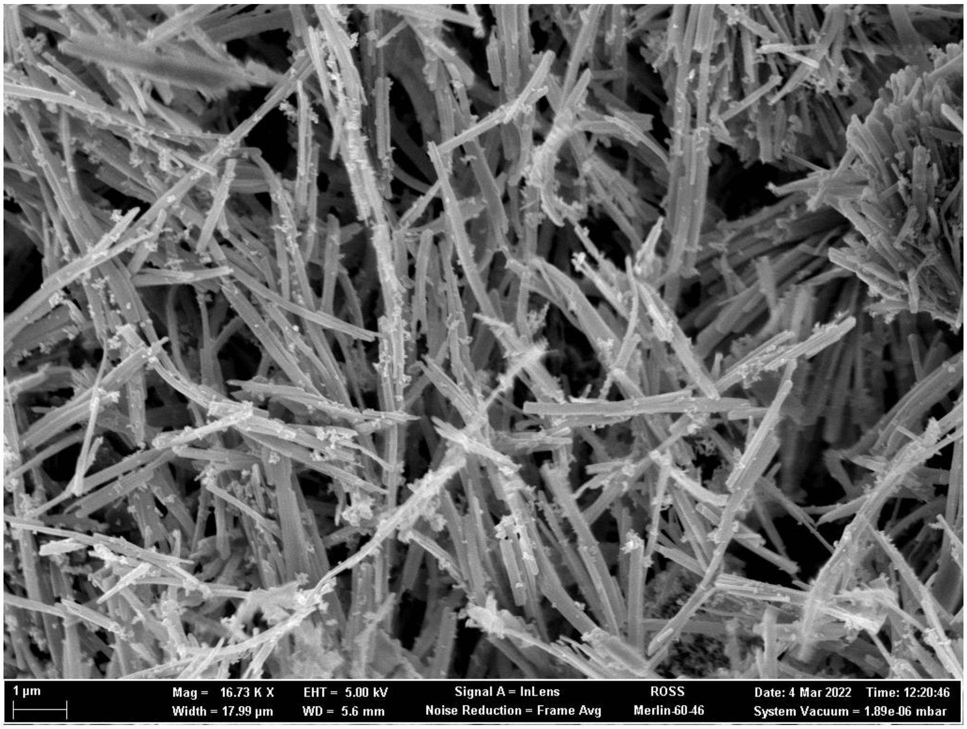

Supplementary Fig. 13. SEM image of microrods from sample ZnO (40 mg).

Supplementary Table 2. Results for experiments varying the concentration of McMT.

Sample	ZnO (mmol)	McMT (mmol)	Molar ratio (ZnO:McMT)	Yield (mg)
McMT (33 mg)	0.25	0.25	1:1	39
McMT (66 mg)	0.25	0.50	1:2	65
McMT (132mg)	0.25	1.00	1:4	69

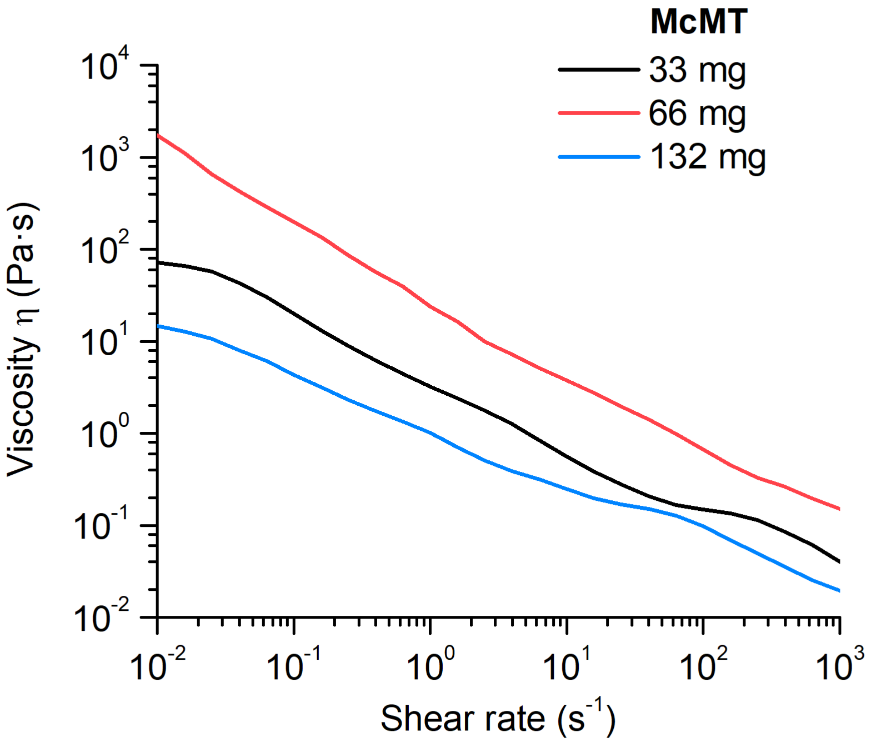

Supplementary Fig. 14. Flow viscosity measurements of the microrod suspensions resulting from experiments varying the concentration of McMT.

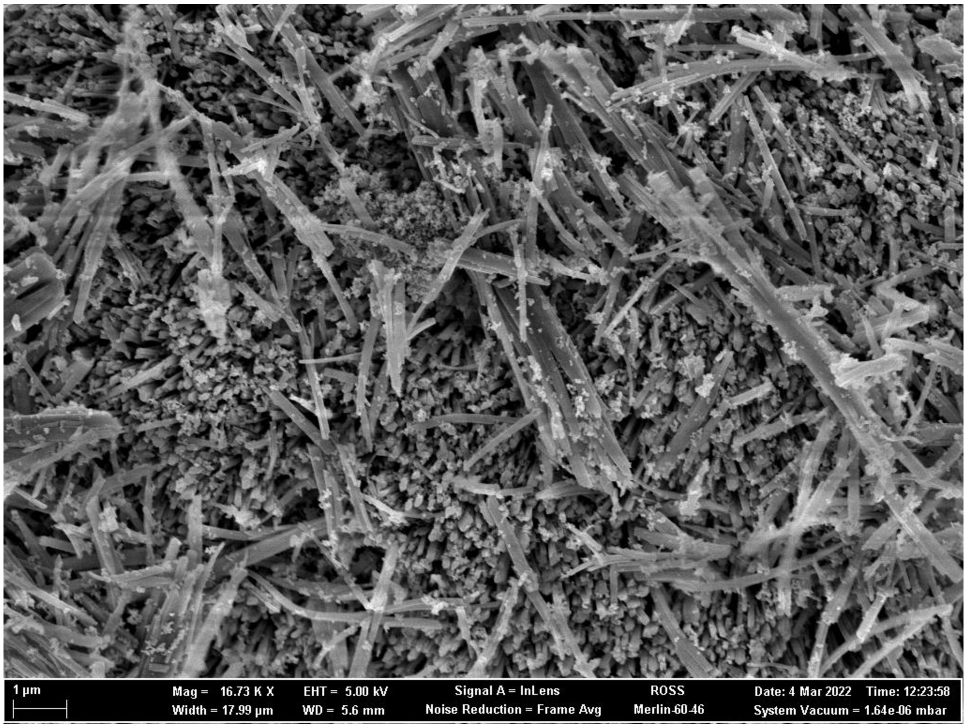

Supplementary Fig. 15. SEM image of microrods from sample McMT (33 mg).

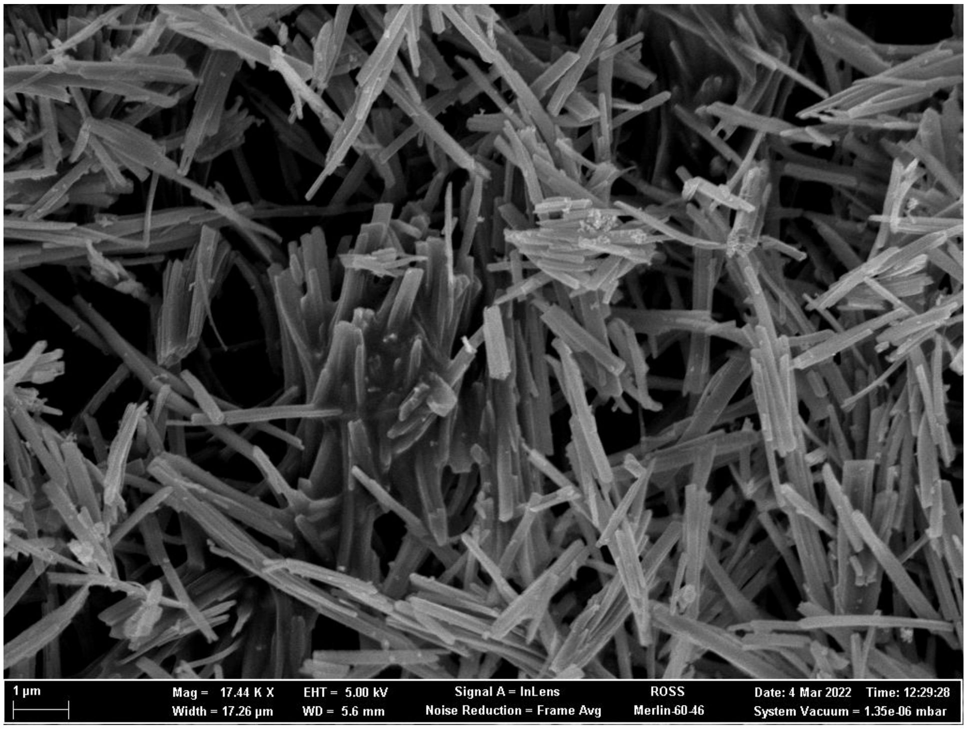

Supplementary Fig. 16. SEM image of microrods from sample McMT (66 mg).

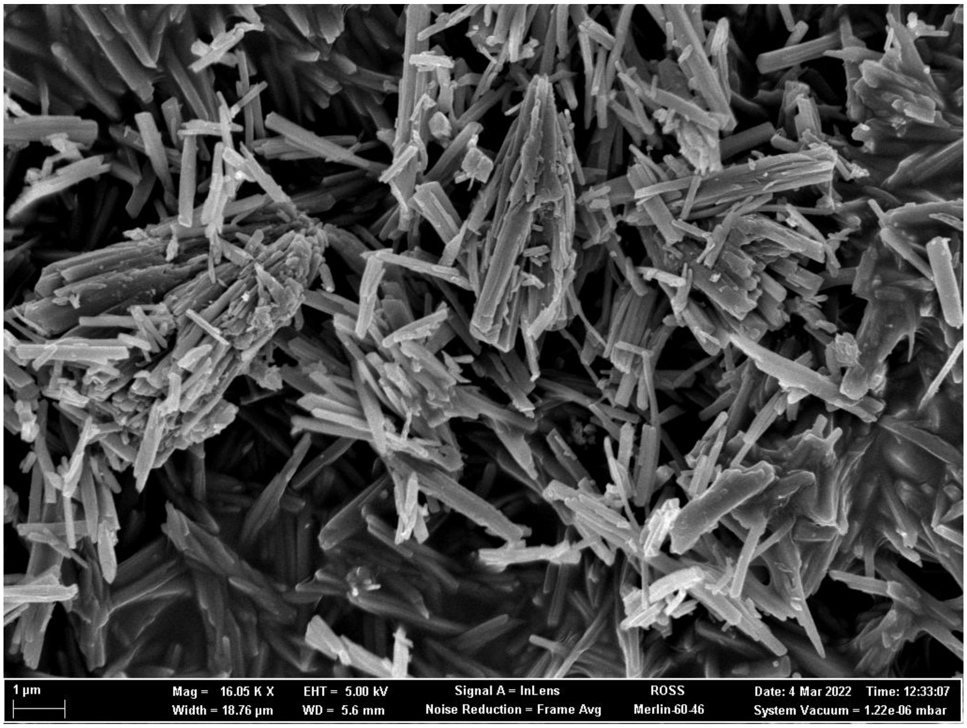

Supplementary Fig. 17. SEM image of microrods from sample McMT (132 mg).

Supplementary Table 3. Particle size analysis for temporal study of the microrod growth.

Reaction time (h)	Yield (mg)	Particle length (μm)	Particle width (μm)	Aspect ratio
0	-	51.3×10^{-3}	-	1*
1	12	2.1	0.2	11
2	42	3.1	0.2	16
4	71	4.3	0.3	14
6	70	4.1	0.3	14

* ZnO nanoparticles are spherical.

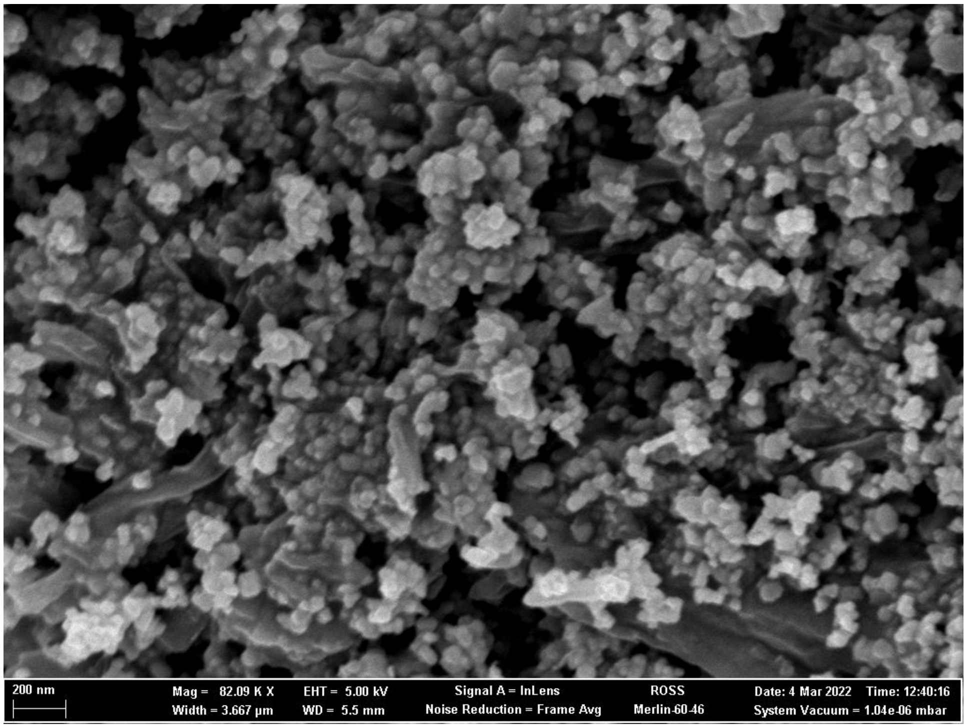

Supplementary Fig. 18. Higher magnification SEM image of microrod growth at 1 h timepoint.

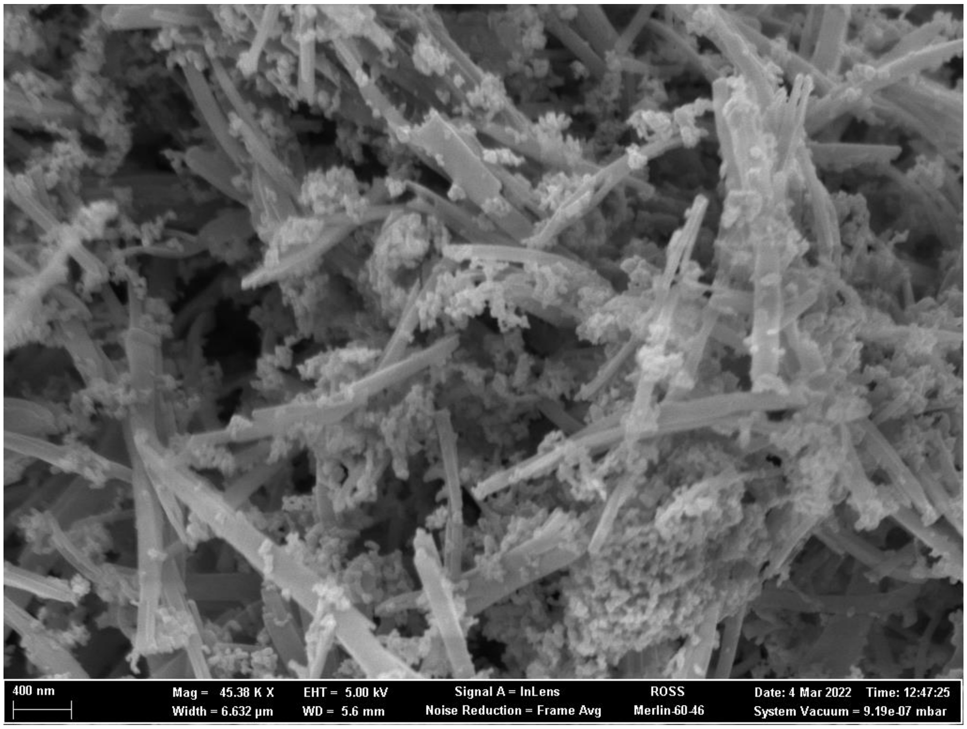

Supplementary Fig. 19. Higher magnification SEM image of microrod growth at 2 h timepoint.

5. Rheology characterization of microrod/PU polymer solution

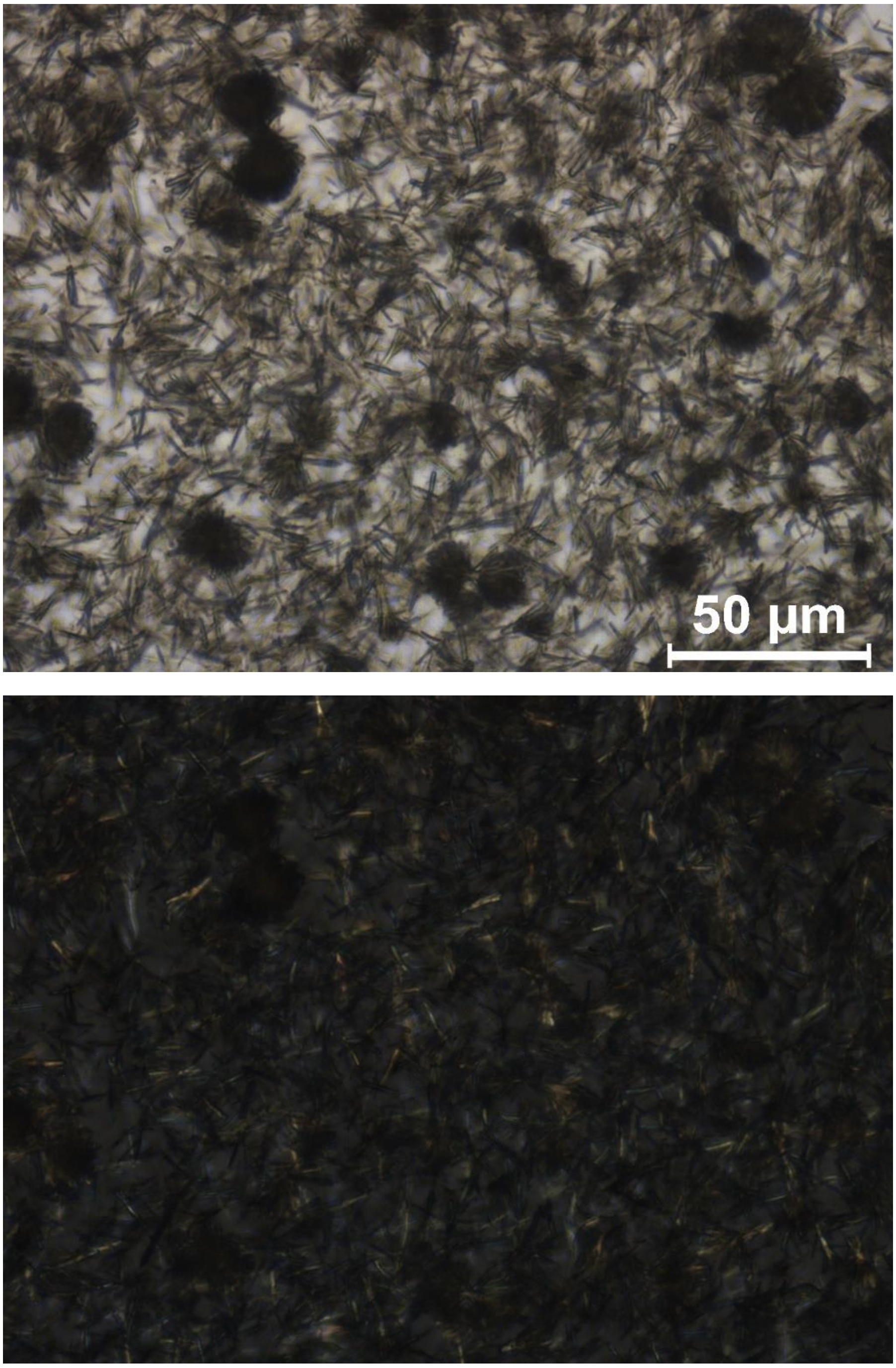

Supplementary Fig. 20. Light microscopy images of the *All components* polymer solution containing the microrods under brightfield (*top*) and cross-polarizer filter (*bottom*).

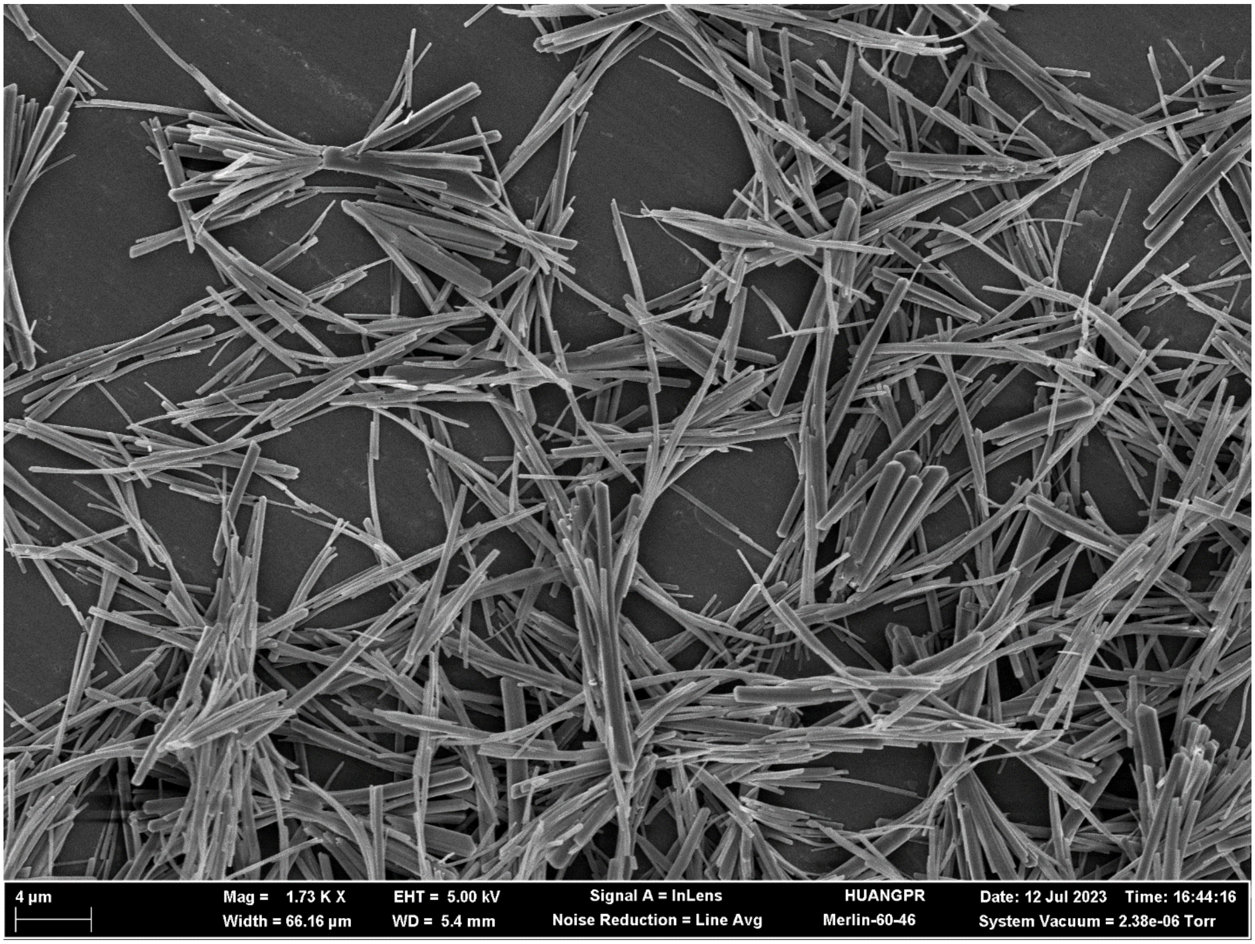

Supplementary Fig. 21. SEM image of the *All components* polymer solution containing the microrods.

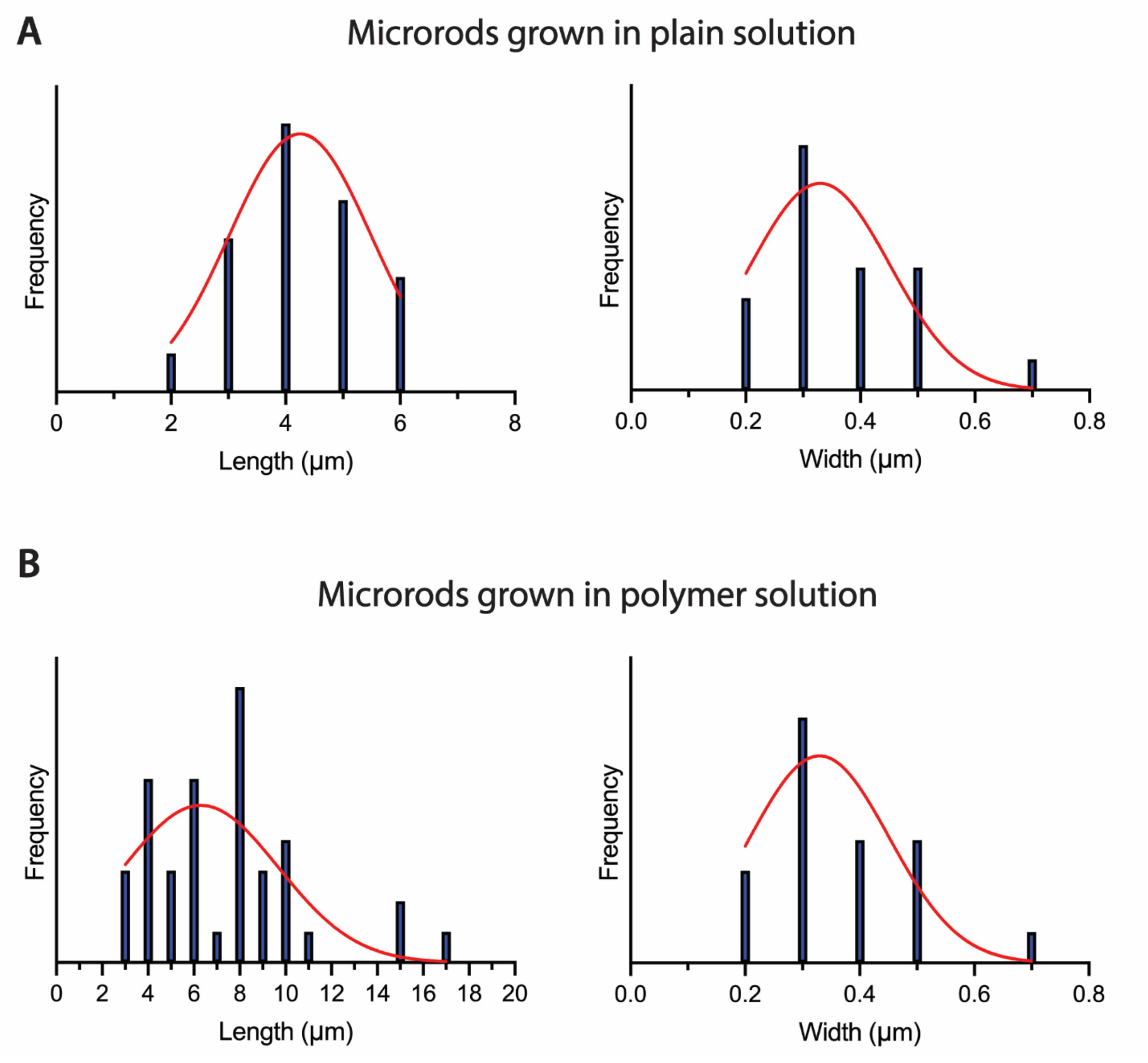

Supplementary Fig. 22. Size distribution graphs (length and width) of microrods grown in (A) plain solution and (B) polyurethane polymer solution.

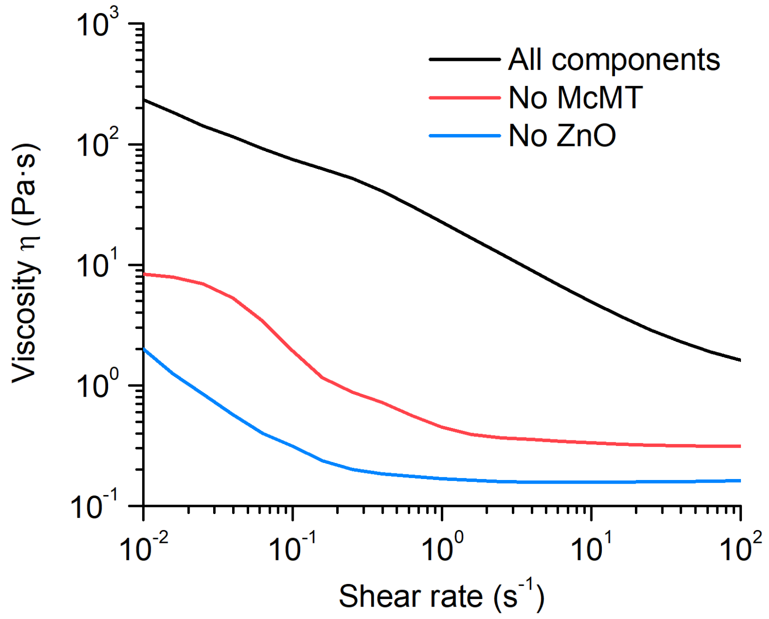

Supplementary Fig. 23. Shear viscosity measurements of microrods grown within polymer organic solution and control reactions (Batch #2).

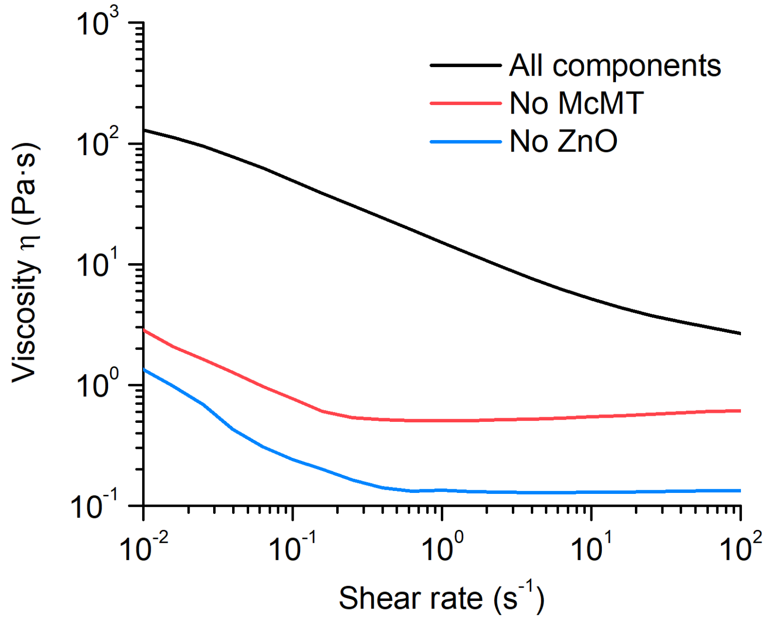

Supplementary Fig. 24. Shear viscosity measurements of microrods grown within polymer organic solution and control reactions (Batch #3).

6. DMA characterization of microrod/azido-PU composites

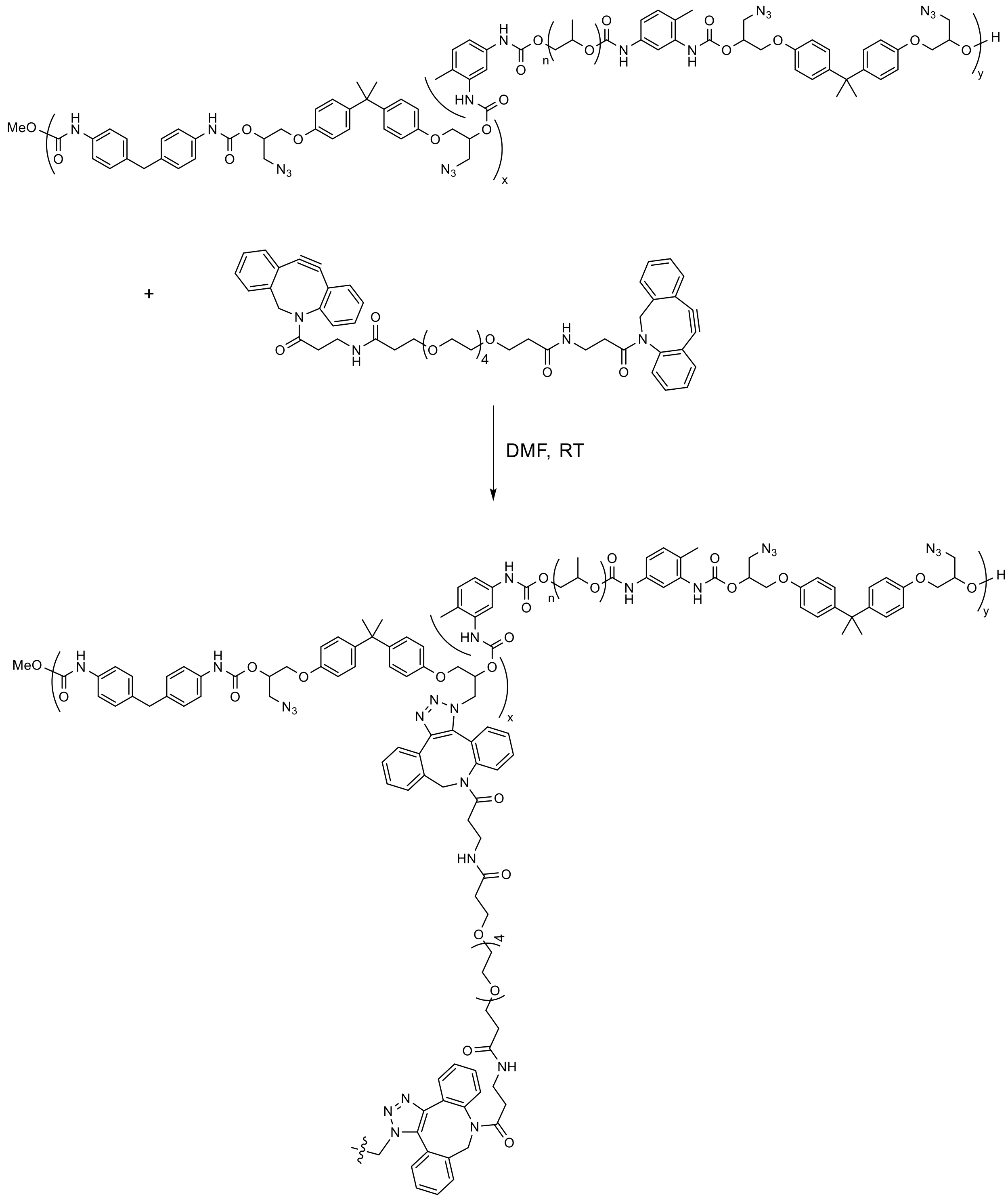

Supplementary Fig. 25. Crosslinking reaction of azido-PU with DBCO-PEG$_5$-DBCO in DMF at room temperature.

<table>
<tr><th>Sample</th><th>Strain sweep
(frequency 1 Hz)</th><th>Frequency sweep
(strain 1 %)</th></tr>
<tr><td>All components</td><td>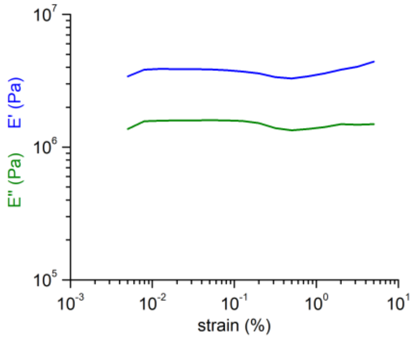
</td><td>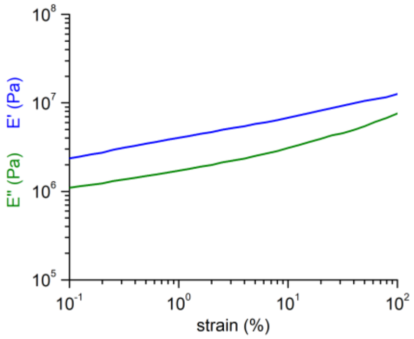
</td></tr>
<tr><td>No McMT</td><td>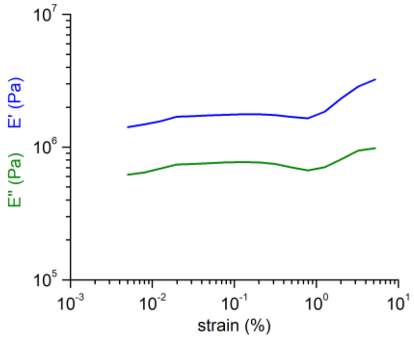
</td><td>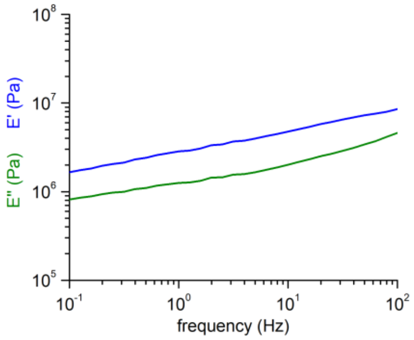
</td></tr>
<tr><td>No ZnO</td><td>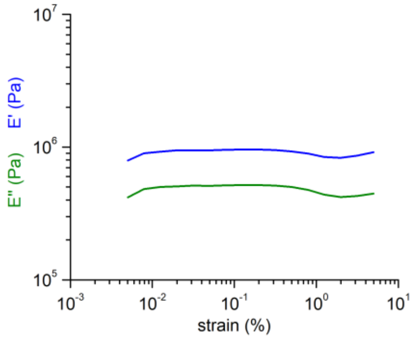
</td><td>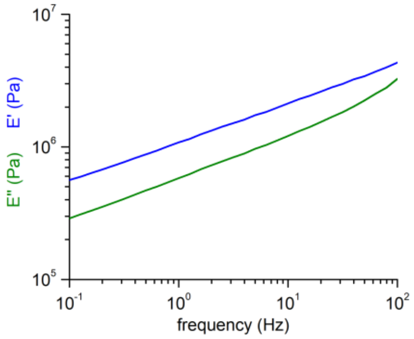
</td></tr>
</table>

Supplementary Fig. 26. Representative compression DMA oscillatory amplitude and frequency measurements of microrod/azido-PU composite and control samples.

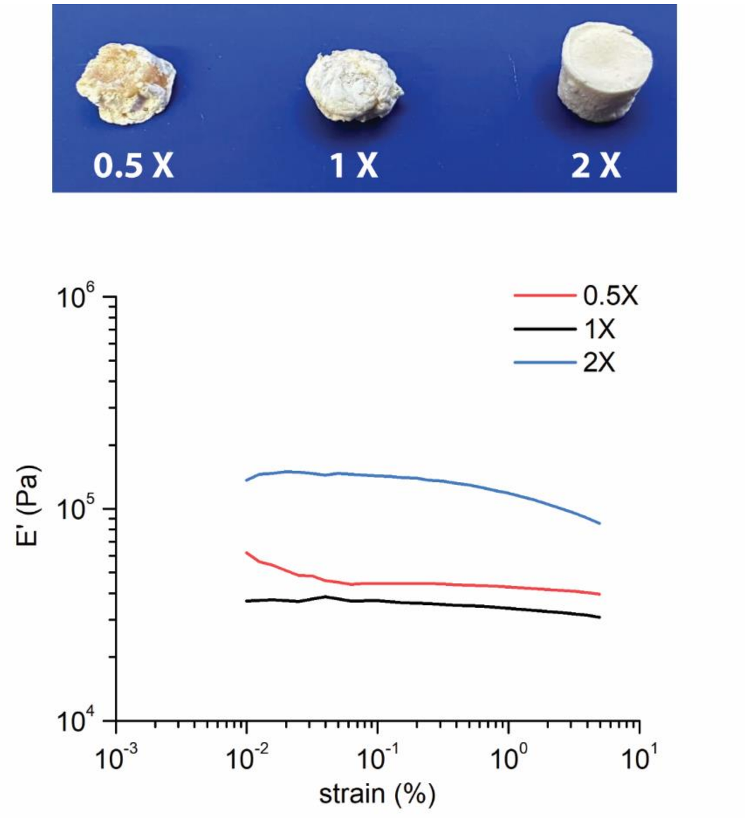

Supplementary Fig. 27. Representative DMA oscillatory amplitude measurements for the azido-PU composite samples with different loadings of microrods.

<table>
<tr><th>Sample</th><th>Strain sweep
(frequency 1 Hz)</th><th>Frequency sweep
(strain 1 %)</th></tr>
<tr><td>0.5 X</td><td>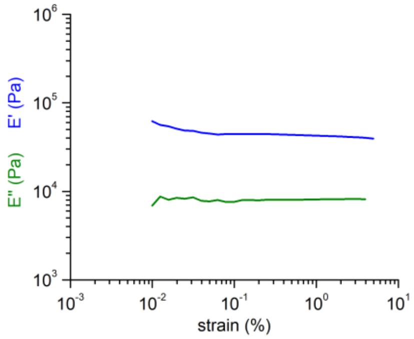
</td><td>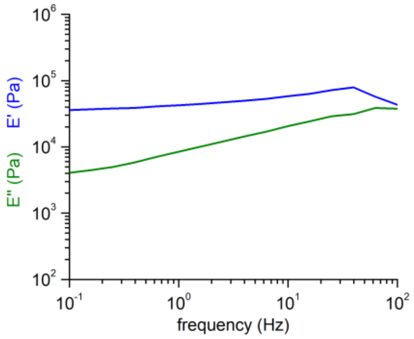
</td></tr>
<tr><td>1 X</td><td>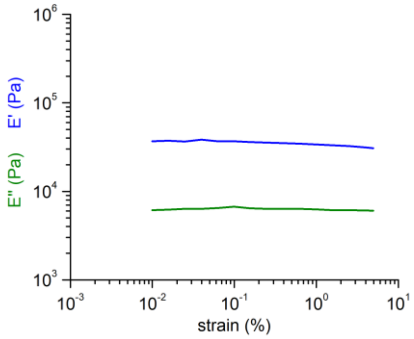
</td><td>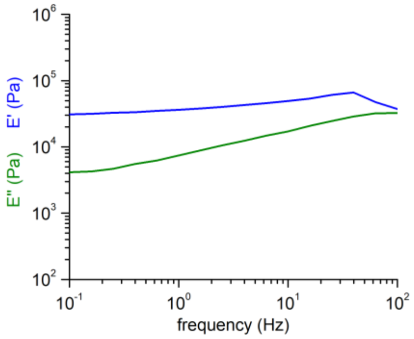
</td></tr>
<tr><td>2 X</td><td>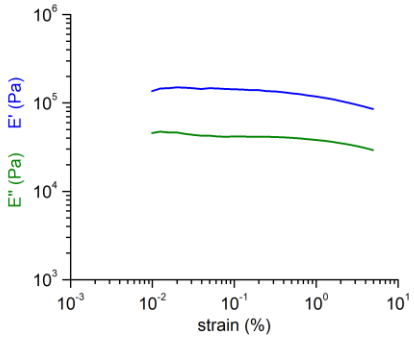
</td><td>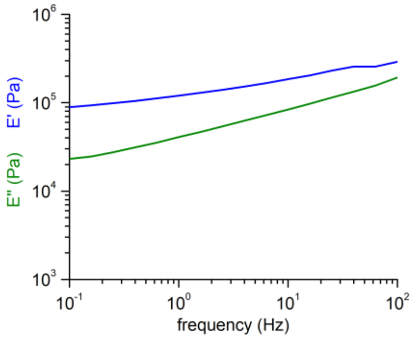
</td></tr>
</table>

Supplementary Fig. 28. Representative compression DMA oscillatory amplitude and frequency measurements of azido-PU composite samples with different loadings of microrods.

7. Images of cross-sections of the microrods/azido-PU composite

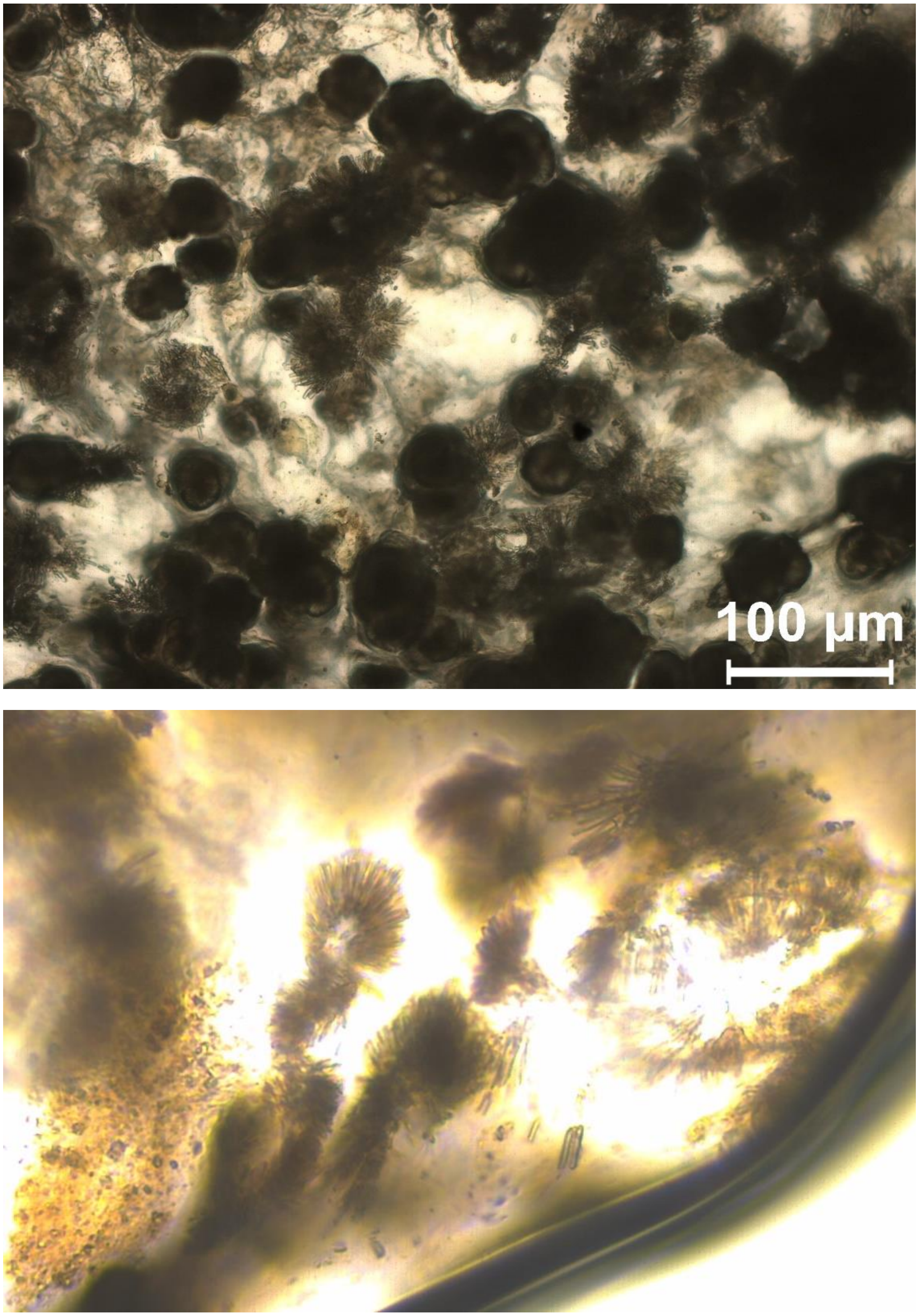

Supplementary Fig. 29. Light microscopy images (bright-field) of the *All components* sample showing the presence of the crystalline microrods in bundles.

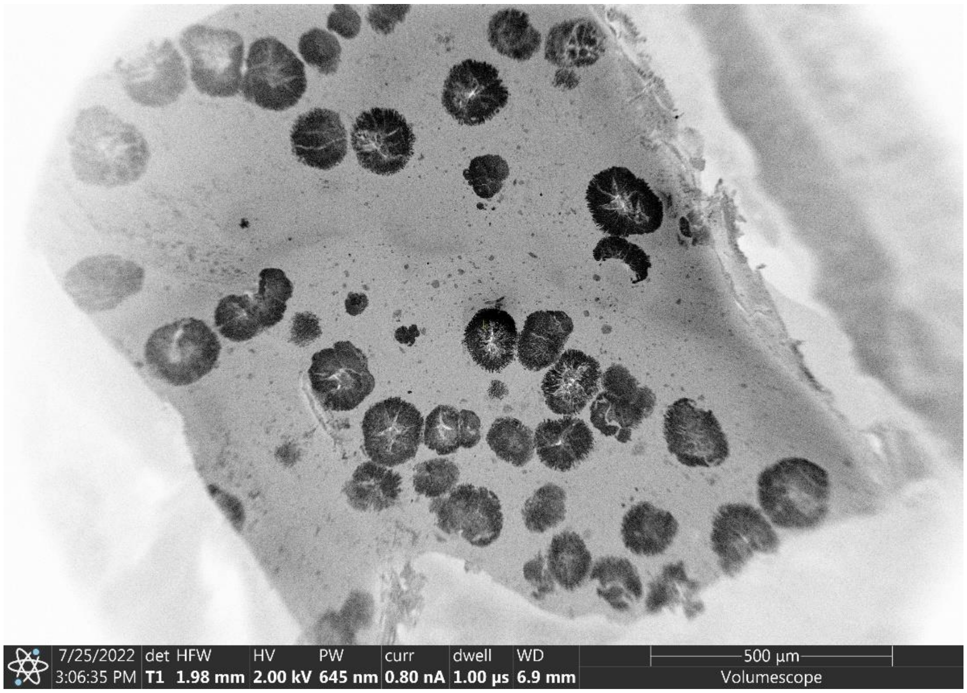

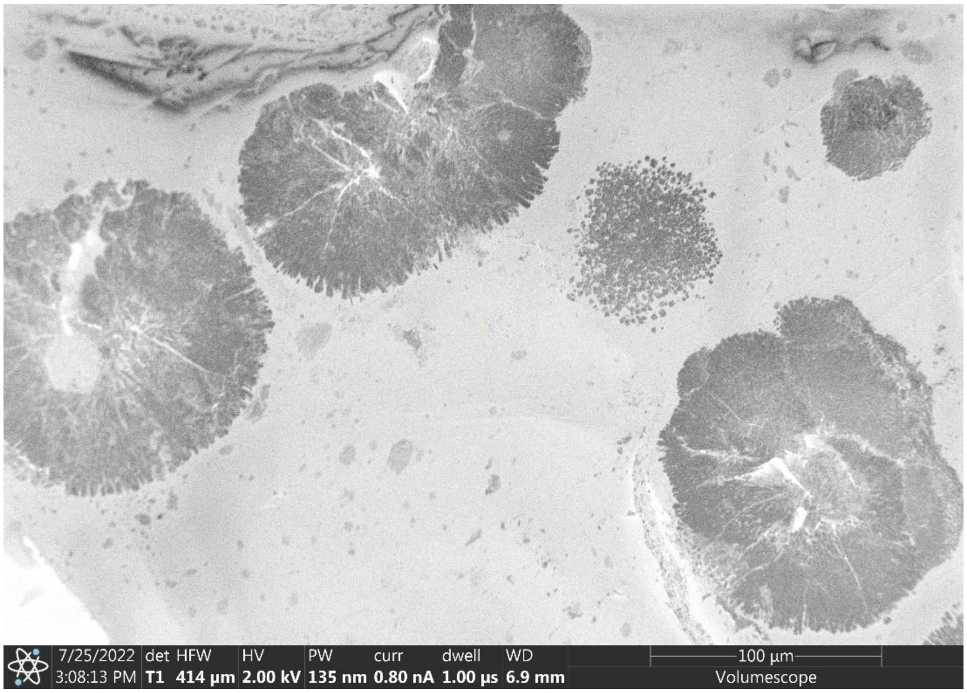

Supplementary Fig. 30. TEM images of microrods in azido-PU matrix.

Supplementary References

(1) Fabretti, A. C.; Peyronel, G.; Franchini, G. C.; Bellei, M. Zinc (II), Cadmium (II) and Mercury (II) Complexes of 2-Methyl-5-Mercapto-1,3,4-Thiadiazole. *Spectrochim. Acta* **1981**, *37*, 587–590.

(2) Tian, H.; Jiang, X.; Yu, Z.; Kloo, L.; Hagfeldt, A.; Sun, L. Efficient Organic-Dye-Sensitized Solar Cells Based on an Iodine-Free Electrolyte. *Angew. Chemie - Int. Ed.* **2010**, *49*, 7328–7331.

(3) Mohapatra, H.; Ayarza, J.; Sanders, E. C.; Scheuermann, A. M.; Griffin, P. J.; Esser-Kahn, A. P. Ultrasound Promoted Step-Growth Polymerization and Polymer Crosslinking Via Copper Catalyzed Azide–Alkyne “Click” Reaction. *Angew. Chemie - Int. Ed.* **2018**, *57*, 11208–11212.
